# Clinically contextualised ECG interpretation: the impact of prior clinical exposure and case vignettes on ECG diagnostic accuracy

**DOI:** 10.1186/s12909-021-02854-x

**Published:** 2021-08-03

**Authors:** Charle André Viljoen, Rob Scott Millar, Kathryn Manning, Julian Hoevelmann, Vanessa Celeste Burch

**Affiliations:** 1 Division of Cardiology, Groote Schuur Hospital, University of Cape Town, Observatory, Cape Town, 7925 South Africa; 2 Department of Medicine, Groote Schuur Hospital, University of Cape Town, Observatory, Cape Town, 7925 South Africa; 3 Cape Heart Institute, University of Cape Town, Observatory, Cape Town, 7925 South Africa; 4 Klinik für Innere Medizin III, Kardiologie, Angiologie und Internistische Intensivmedizin, Universitätsklinikum des Saarlandes, Saarland University Hospital, Homburg/Saar, Germany

**Keywords:** Electrocardiography, ECG, Experiential learning, Clinical context, Case vignette

## Abstract

**Background:**

ECGs are often taught without clinical context. However, in the clinical setting, ECGs are rarely interpreted without knowing the clinical presentation. We aimed to determine whether ECG diagnostic accuracy was influenced by knowledge of the clinical context and/or prior clinical exposure to the ECG diagnosis.

**Methods:**

Fourth- (junior) and sixth-year (senior) medical students, as well as medical residents were invited to complete two multiple-choice question (MCQ) tests and a survey. Test 1 comprised 25 ECGs without case vignettes. Test 2, completed immediately thereafter, comprised the same 25 ECGs and MCQs, but with case vignettes for each ECG. Subsequently, participants indicated in the survey when last, during prior clinical clerkships, they have seen each of the 25 conditions tested. Eligible participants completed both tests and survey. We estimated that a minimum sample size of 165 participants would provide 80% power to detect a mean difference of 7% in test scores, considering a type 1 error of 5%.

**Results:**

This study comprised 176 participants (67 [38.1%] junior students, 55 [31.3%] senior students, 54 [30.7%] residents). Prior ECG exposure depended on their level of training, i.e., junior students were exposed to 52% of the conditions tested, senior students 63.4% and residents 96.9%. Overall, there was a marginal improvement in ECG diagnostic accuracy when the clinical context was known (Cohen’s *d* = 0.35, *p* < 0.001). Gains in diagnostic accuracy were more pronounced amongst residents (Cohen’s *d* = 0.59, *p* < 0.001), than senior (Cohen’s *d* = 0.38, *p* < 0.001) or junior students (Cohen’s *d* = 0.29, *p* < 0.001). All participants were more likely to make a correct ECG diagnosis if they reported having seen the condition during prior clinical training, whether they were provided with a case vignette (odds ratio [OR] 1.46, 95% confidence interval [CI] 1.24–1.71) or not (OR 1.58, 95% CI 1.35–1.84).

**Conclusion:**

ECG interpretation using clinical vignettes devoid of real patient experiences does not appear to have as great an impact on ECG diagnostic accuracy as prior clinical exposure. However, exposure to ECGs during clinical training is largely opportunistic and haphazard. ECG training should therefore not rely on experiential learning alone, but instead be supplemented by other formal methods of instruction.

**Supplementary Information:**

The online version contains supplementary material available at 10.1186/s12909-021-02854-x.

## Introduction

The electrocardiogram (ECG) is the most frequently used investigation to diagnose and monitor cardiac disease [1]. Even after more than 120 years of use in clinical practice [2], there is no better investigation for the detection arrhythmias and conduction disturbances [3, 4]. Contemporary guidelines recommend an urgent ECG in any patient presenting with chest pain or suspected of having a myocardial ischaemia [5–7]. Although the ECG is a powerful tool in diagnosing heart disease, incorrect ECG interpretation can lead to inappropriate clinical decisions with adverse outcomes [8–10].

Over the past two decades, lack of ECG competence has been well described for medical students [11–14], residents [15–23], and qualified clinicians worldwide [24–29]. Undergraduate [30–32] and postgraduate ECG curricula [33–38] have been proposed in an attempt to standardise training in Electrocardiography. While formal ECG teaching is predetermined and predictable [30], ECG learning based on real patient encounters is opportunistic and unpredictable. Furthermore, to the best of our knowledge, this type of ECG learning during clinical clerkships or residency programmes is poorly quantified and its influence on the diagnostic accuracy of ECG interpretation is not known.

In other domains of Medicine, such as Dermatology and Radiology, where visual stimuli are also central to the diagnostic process, research has shown that knowing the clinical context was associated with improved diagnostic accuracy [39, 40]. However, prior studies evaluating the impact of case vignettes on diagnostic accuracy in Electrocardiography *per se* have yielded conflicting results. Grum et al. showed the provision of clinical scenarios did not influence the accuracy of ECG interpretation of third year medical students [41]. However, third year students typically have little clinical experience compared to more senior trainees. On the contrary, Hatala et al. found that clinical scenarios were helpful in ECG interpretation [42, 43]. They showed that provision of the clinical context was more helpful in trainees with greater clinical experience, i.e. residents as compared to graduating medical students [42, 43]. However, Wood et al., found that ECG diagnostic accuracy of medical students and qualified emergency physicians was not influenced by the provision of clinical vignettes. Nevertheless, the authors established by means of eye tracking technology that qualified clinicians were faster to identify the relevant ECG leads that displayed abnormal waveforms in support of an ECG diagnosis. As expected, qualified emergency physicians also showed better ECG diagnostic accuracy than medical students [44]. Two significant limitations of the existing literature are the small sample size of published work and the limited range of ECG diagnoses evaluated. Moreover, it was not apparent in these studies what the extent of exposure to ECGs was during undergraduate clinical clerkships or postgraduate residency programmes.

The aim of this study was to determine the accuracy of ECG interpretation of medical trainees with different levels of clinical experience with or without the aid of a clinical vignette. In addition, this study set out to evaluate whether these trainees demonstrated greater ECG diagnostic accuracy if they had prior exposure to patients with ECG abnormalities that are considered core knowledge for medical training [30]. This study intended to advance on earlier work [41–44], by recruiting more participants, from both undergraduate and postgraduate training programmes, and using an expanded set of ECG diagnoses, including both waveform and rhythm disturbances.

## Methods

### Participants

We performed a cross-sectional study on undergraduate and postgraduate students from the University of Cape Town (UCT). The undergraduate trainees comprised fourth- and sixth-year medical students at the end of their Internal Medicine clerkship (enrolled in 2017). The postgraduate trainees were residents from the Department of Medicine (enrolled between 2018 and 2020), with at least 4 years of working experience after graduating as medical doctors. Participation was voluntary.

### Formal ECG training

At the University of Cape Town, medical students are introduced to the basic principles of Electrocardiography during a series of lectures in their third year of study. Training in Electrocardiography continues during the fourth-year and sixth-year Internal Medicine clinical clerkships in the form of lectures. The lectures cover a core syllabus of ECG diagnoses [30], which include arrhythmias (sinus arrhythmia, sinus arrest with escape rhythm, first degree AV block, Mobitz type I and II second degree AV block, third degree AV block, atrial fibrillation [AF] with normal and uncontrolled rate, atrial flutter, AV node re-entrant tachycardia [AVNRT], ventricular tachycardia [VT] and ventricular fibrillation [VF]) and waveform abnormalities (left and right atrial enlargement, left ventricular hypertrophy [LVH], right ventricular hypertrophy [RVH], left bundle branch block [LBBB], right bundle branch block [RBBB], left anterior fascicular block [LAFB], Wolff-Parkinson-White [WPW] pattern, ST-segment elevation myocardial infarction [STEMI], pericarditis, hyperkalaemia, long QT syndrome). Over and above lectures, students are required to analyse and interpret ECGs of patients whom they encounter during their clinical clerkships. Post-graduate training encompasses formal lectures and ECG tutorials during clinical training, in addition to analysing and interpreting ECGs of patients for whom they provide care.

### Study design

**Fig. 1:**
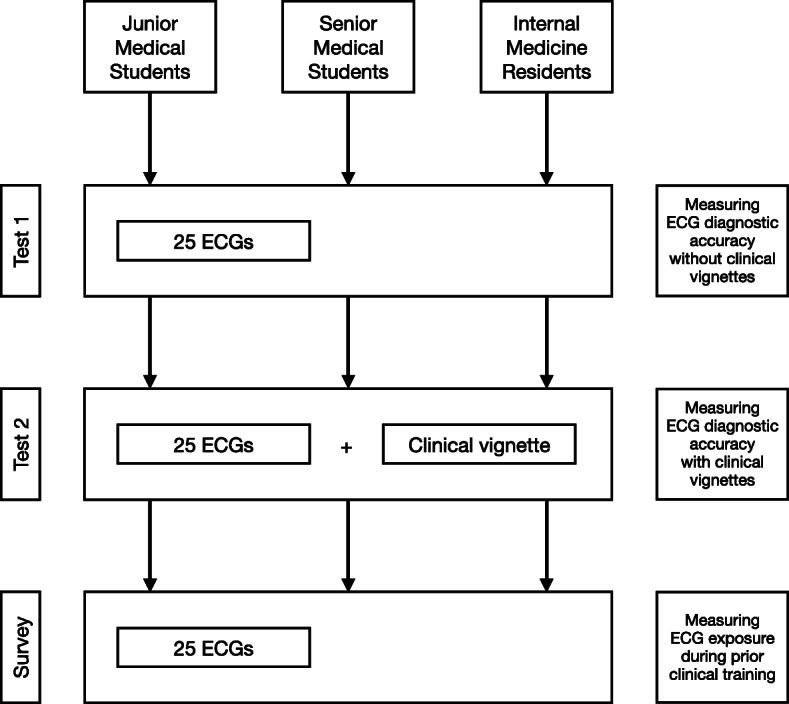
Study flow. The 25 ECGs included in the two tests and survey were twelve rhythm abnormalities (sinus arrhythmia, sinus arrest with escape rhythm, first degree AV block, Mobitz type I and II second degree AV block, third degree AV block, atrial fibrillation [AF] with normal and uncontrolled rate, atrial flutter, AV node re-entrant tachycardia [AVNRT], ventricular tachycardia [VT] and ventricular fibrillation [VF]), and thirteen waveform abnormalities (left and right atrial enlargement, left ventricular hypertrophy [LVH], right ventricular hypertrophy [RVH], left bundle branch block [LBBB], right bundle branch block [RBBB], left anterior fascicular block [LAFB], Wolff-Parkinson-White [WPW] pattern, anterior and inferior ST-segment elevation myocardial infarction [STEMI], pericarditis, hyperkalaemia, long QT syndrome)

**Fig. 2:**
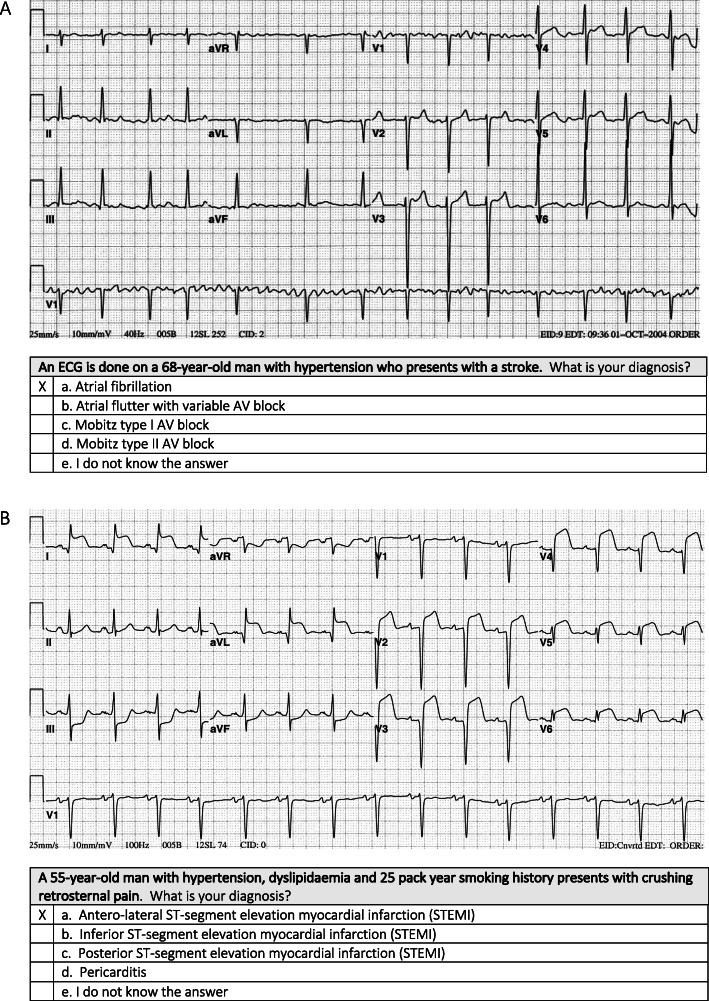
An example of the ECG, case vignette and multiple-choice questions asked for **A** rhythm abnormalities and **B** waveform abnormalities. Case vignettes were only shown in Test 2

The study flow is outlined in Fig. 1. All participants completed two tests and a survey, on the same day, in the following order:

- During Test 1, participants were shown 25 ECGs. Each ECG was accompanied by a multiple-choice question (MCQ). For each question, there were five optional answers – four possible diagnoses (of which only one was correct), and a fifth option, i.e. “I am not sure what the answer is”. *Test 1 measured accuracy of ECG diagnoses in the absence of a case vignette.*
- Immediately after submission of Test 1, participants completed Test 2. During Test 2, participants were shown the same 25 ECGs, which were accompanied by a case vignette (Fig. 2). They were provided with the same MCQs and the same five optional answers as in Test 1. *Test 2 measured the accuracy of ECG diagnoses in the presence of a case vignette.* The case vignettes described the patient demographic details (e.g., age and gender), common comorbidities associated with the condition (where appropriate) and typical clinical presentation to the emergency unit, ward or outpatient department, where the particular ECG was done.
- Once Test 2 was submitted, participants completed a survey, which asked them to indicate when last during their prior clinical rotations they have seen the 25 ECGs that were included in Test 1 and Test 2. *The survey measured exposure to a core curriculum of ECGs during prior clinical training.*

### Assessment of ECG diagnostic accuracy

The invigilated, password protected MCQ tests and survey were administered at the computer laboratories at UCT’s Faculty of Health Sciences. The 25 topics covered in the MCQ tests were considered to be core knowledge for undergraduate medical training at our institution [30]. Of these, twelve were rhythm abnormalities (sinus arrhythmia, sinus arrest with escape rhythm, first degree AV block, Mobitz type I and II second degree AV block, third degree AV block, AF with normal and uncontrolled rate, atrial flutter, AVNRT, VT and VF), and thirteen were waveform abnormalities (left and right atrial enlargement, LVH, RVH, LBBB, RBBB, LAFB, WPW pattern, anterior and inferior STEMI, pericarditis, hyperkalaemia, long QT syndrome). The ECGs and their answers are included in the Supplementary material.

The investigators of this study (three specialist physicians with a special interest in Electrocardiography) agreed that the ECGs used in the tests were unequivocal examples of the conditions tested, and that the questions and multiple-choice options were fair for the given ECGs. The clinical scenarios described for each ECG provided the typical presentation of the particular condition. The ECGs included in this study have been validated in a Delphi study, in which the priorities for undergraduate ECG learning were determined [30].

Participants were allowed 30 min to complete each test. Each correct answer was awarded one mark and negative marking was not applied. The answers to the questions were only made available to the students after completion of both tests. The results of the MCQ tests in this study did not contribute to the participants’ course marks.

### Estimated sample size needed for an adequately powered study

We estimated that a minimum sample size of 165 participants would provide 80% power to detect a mean difference of 7% in the test scores with and without case vignettes, considering an α (type 1 error) of 5%. This calculation was based on the results of previous studies assessing the impact of case vignettes on ECG diagnostic accuracy amongst medical students and residents [41, 43].

### Eligibility to be included in the study

Participants were only included if they completed both Test 1 and Test 2, as well as the survey on prior exposure to ECGs during clinical training. Students were enrolled on the last day of their Internal Medicine clerkship, during the second half of the academic year. Residents took part in the study during dedicated postgraduate training time, provided that they had worked more than half a year in our institution.

### Statistical analysis

Statistical analyses were performed on anonymised data using Stata (Version 14.2, StataCorp, College Station TX, USA). Descriptive statistics were used to summarise the ECG test scores and when last ECGs were seen during prior clinical training. For each ECG analysed, the proportion of correct answers for each cohort was calculated by the numerator/denominator and expressed as percentages. Within group change from Test 1 to Test 2 was analysed using McNemar’s test (comparing diagnostic accuracy for each ECG in Test 1 and Test 2) and the signed-rank test (comparing total scores in Test 1 and Test 2). Cohen’s *d* was used to determine the effect size (practical significance) of the differences in total test scores, with values of 0.2, 0.5 and 0.8 indicating small, moderate and large effect sizes respectively. Associations between correct ECG diagnoses (with or without the provision of a case vignette) and whether the ECGs were seen during prior clinical training were assessed using odds ratios (OR), as determined by logistic regression. Where applicable, a *p* value of < 0.05 was considered statistically significant and 95% confidence intervals (CI) were used to determine the precision of estimates.

## Results

This study comprised 176 participants, of which 67 (38.1%) were junior students, 55 (31.3%) were senior students and 54 (30.7%) were medical residents.

### Exposure to ECGs during clinical training

**Fig. 3:**
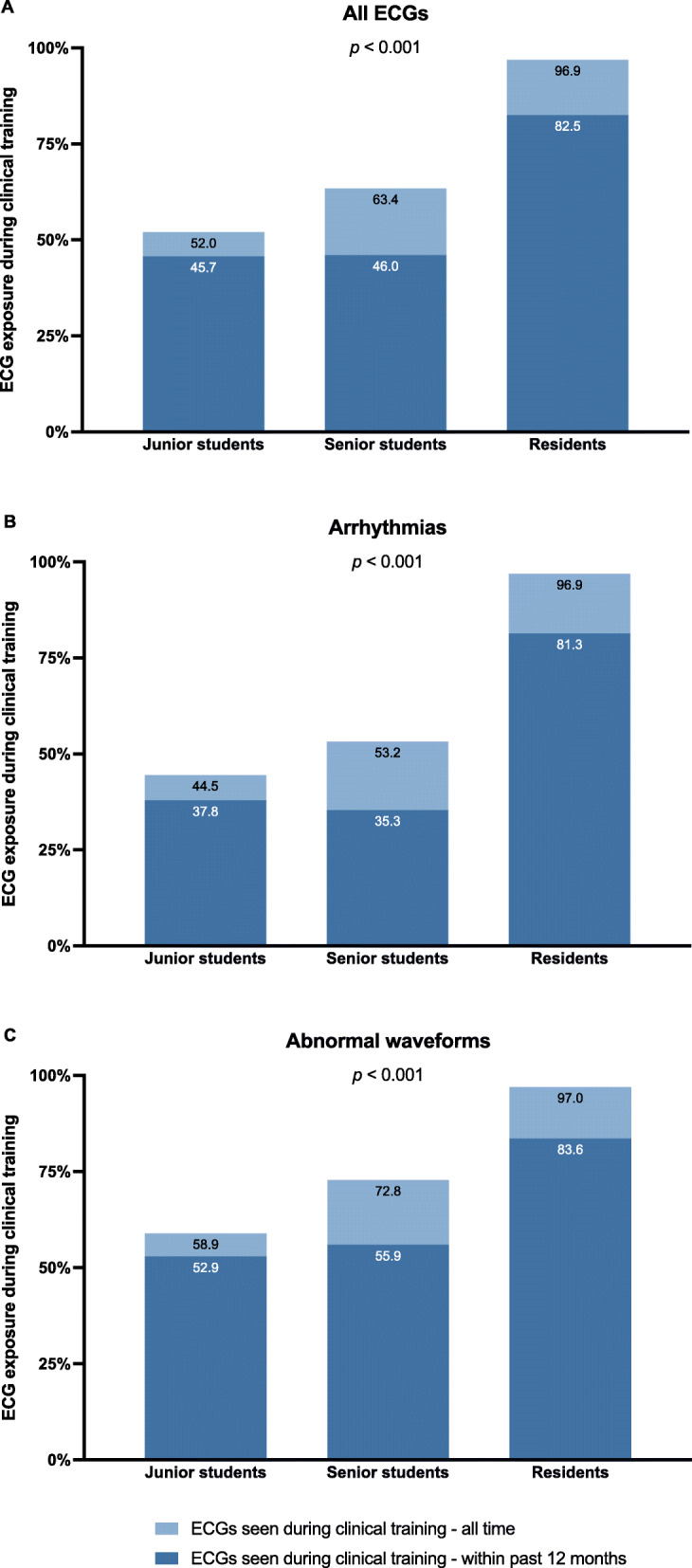
Exposure to ECGs during clinical training, for **A** all ECGs tested, **B** arrhythmias and **C** waveform abnormalities

As illustrated in Fig. 3, junior medical students were exposed to just over half of the 25 ECGs in the tests (i.e., core ECG curriculum) by the end of their clinical clerkship, whereas senior medical students reported having seen about two thirds of these ECGs before graduating. Medical residents were exposed to almost all the ECGs during their clinical training. For all participants, most conditions were seen within 12 months prior to the study. Both junior and senior medical students were exposed to less arrhythmias during their clinical clerkships than ECGs with abnormal waveforms. AV blocks and ventricular arrhythmias were not frequently encountered by junior students (Mobitz type I second degree AV block [Wenckebach] 29.9%, Mobitz type II second degree AV block 31.3%, third degree AV block 28.4%, VT 47.8%, VF 32.8%). Just more than half of the senior students were reportedly exposed to third degree AV block (56.4%) and VT (50.9%) prior to graduation, but few reported prior exposure to Mobitz type I second degree AV block (Wenckebach, 45.5%), Mobitz type II second degree AV block (36.4%) or VF (38.2%). Few medical students were exposed to patients with AVNRT (junior 19.4%, senior 20%) or WPW (junior 14.9%, senior 21.8%). Table 1 provides a detailed breakdown of which conditions each cohort had previously been exposed to during their clinical training.

**Table. 1:** ECGs reportedly seen during prior clinical training

	Junior students	Senior students	Residents	***p*** value
	*n* = 67	*n* = 55	*n* = 54	
**Arrhythmias**				
Sinus arrhythmia	54 (80.6)	50 (90.9)	51 (94.4)	0.048
Sinus arrest	13 (19.4)	19 (34.5)	49 (90.7)	< 0.001
First degree AV block	31 (46.3)	30 (54.5)	53 (98.1)	< 0.001
Mobitz type I second degree AV block (Wenckebach)	20 (29.9)	25 (45.5)	53 (98.1)	< 0.001
Mobitz type II second degree AV block	21 (31.3)	20 (36.4)	52 (96.3)	< 0.001
Third degree AV block	19 (28.4)	31 (56.4)	52 (96.3)	< 0.001
Atrial fibrillation with normal rate	57 (85.1)	47 (85.5)	54 (100.0)	0.012
Atrial fibrillation with uncontrolled rate	42 (62.7)	47 (85.5)	54 (100.0)	< 0.001
Atrial flutter	34 (50.7)	22 (40.0)	52 (96.3)	< 0.001
AV nodal re-entrant tachycardia	13 (19.4)	11 (20.0)	52 (96.3)	< 0.001
Ventricular tachycardia	32 (47.8)	28 (50.9)	53 (98.1)	< 0.001
Ventricular fibrillation	22 (32.8)	21 (38.2)	53 (98.1)	< 0.001
**Waveform abnormalities**				
Left anterior fascicular block	21 (31.3)	21 (38.2)	49 (90.7)	< 0.001
Left bundle branch block	45 (67.2)	50 (90.9)	54 (100.0)	< 0.001
Right bundle branch block	38 (56.7)	45 (81.8)	52 (96.3)	< 0.001
Wolff-Parkinson-White pattern	10 (14.9)	12 (21.8)	49 (90.7)	< 0.001
Left atrial enlargement	45 (67.2)	48 (87.3)	52 (96.3)	< 0.001
Right atrial enlargement	43 (64.2)	49 (89.1)	52 (96.3)	< 0.001
Left ventricular hypertrophy	63 (94.0)	55 (100.0)	54 (100.0)	0.036
Right ventricular hypertrophy	47 (70.1)	49 (89.1)	53 (98.1)	< 0.001
Anterior STEMI	56 (83.6)	54 (98.2)	54 (100.0)	< 0.001
Inferior STEMI	58 (86.6)	52 (94.5)	53 (98.1)	0.043
Pericarditis	38 (56.7)	30 (54.5)	53 (98.1)	< 0.001
Hyperkalaemia	29 (43.3)	31 (56.4)	53 (98.1)	< 0.001
Prolonged QT interval	20 (29.9)	25 (45.5)	53 (98.1)	< 0.001

Values are N (%). *AV* atrioventricular, *STEMI *ST-segment elevation myocardial infarction

### Accuracy of ECG interpretation with or without a case vignette

**Fig. 4:**
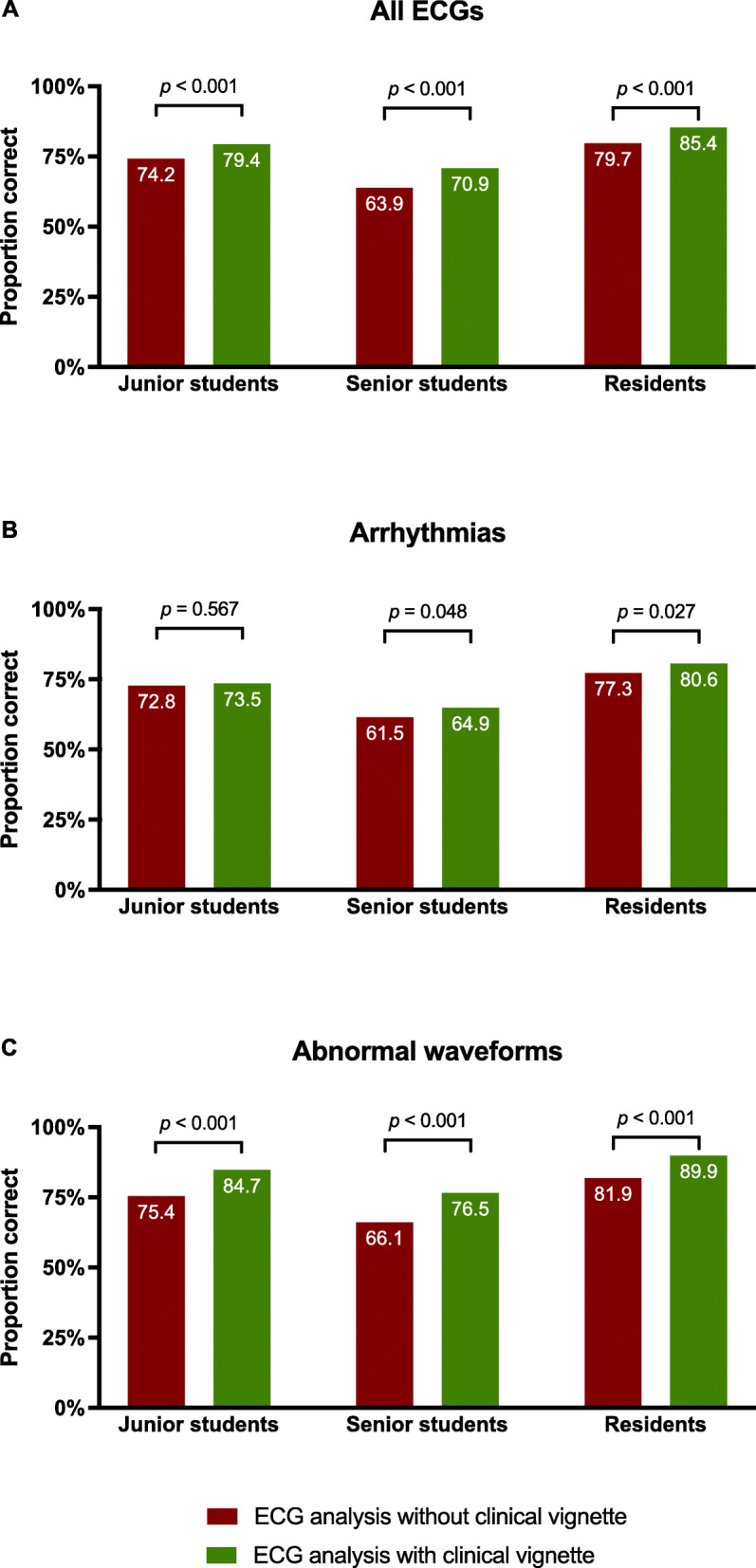
Difference of ECG diagnostic accuracy when participants were provided with a case vignette or not, for **A** all ECGs tested, **B** arrhythmias and **C** waveform abnormalities

Figure 4A demonstrates the proportion of ECGs that were correctly analysed when participants were provided with a case vignette or not. Overall, all groups showed a marginal improvement in accuracy of ECG interpretation when the clinical context was known to them (Cohen’s *d* = 0.35, *p* < 0.001). The gains in accuracy were more pronounced amongst the residents (Cohen’s *d* = 0.59, *p* < 0.001), than amongst senior (Cohen’s *d* = 0.38, *p* < 0.001) or junior students (Cohen’s *d* = 0.29, *p* < 0.001).

As shown in Fig. 4B, subgroup analyses showed that junior students were not influenced by case vignettes when interpreting arrhythmias. Although statistically significant, senior students and residents showed an increase of only 3 percentage units in their scores of the arrhythmia section of the tests, when they were provided with case vignettes. All groups showed an increase in diagnostic accuracy when they were provided with a case vignette for ECGs with abnormal waveforms (Fig. 4C). Table 2 provides a detailed breakdown of how each cohort’s ECG diagnostic accuracy was influenced by whether they were provided with a case vignette or not at the time of ECG analysis.

**Table. 2:** Proportion of ECGs correctly diagnosed when participants were provided with a case vignette or not

	Junior students			Senior students			Residents		
	*n* = 67			*n* = 55			*n* = 54		
	Without scenario	With scenario	*p*-value	Without scenario	With scenario	*p*-value	Without scenario	With scenario	*p*-value
**Arrhythmias**									
Sinus arrhythmia	59 (88.1)	59 (88.1)	1	41 (74.5)	47 (85.5)	0.083	51 (94.4)	49 (90.7)	0.317
Sinus arrest	55 (82.1)	56 (83.6)	0.706	39 (70.9)	44 (80.0)	0.096	43 (79.6)	43 (79.6)	1
First degree AV block	54 (80.6)	49 (73.1)	0.059	38 (69.1)	40 (72.7)	0.527	47 (87.0)	51 (94.4)	0.103
Mobitz type I second degree AV block	49 (73.1)	49 (73.1)	1	30 (54.5)	29 (52.7)	0.781	32 (59.3)	29 (53.7)	0.317
Mobitz type II second degree AV block	54 (80.6)	52 (77.6)	0.480	33 (60.0)	37 (67.3)	0.317	45 (83.3)	40 (74.1)	0.059
Third degree AV block	57 (85.1)	54 (80.6)	0.257	38 (69.1)	39 (70.9)	0.563	51 (94.4)	50 (92.6)	0.654
Atrial fibrillation with normal rate	56 (83.6)	58 (86.6)	0.414	36 (65.5)	40 (72.7)	0.103	47 (87.0)	51 (94.4)	0.103
Atrial fibrillation with uncontrolled rate	49 (73.1)	45 (67.2)	0.248	33 (60.0)	28 (50.9)	0.197	30 (55.6)	41 (75.9)	0.002
Atrial flutter	43 (64.2)	41 (61.2)	0.593	30 (54.5)	35 (63.6)	0.197	36 (66.7)	36 (66.7)	1
AV nodal re-entrant tachycardia	40 (59.7)	50 (74.6)	0.012	27 (49.1)	32 (58.2)	0.096	44 (81.5)	49 (90.7)	0.059
Ventricular tachycardia	39 (58.2)	42 (62.7)	0.257	34 (61.8)	33 (60.0)	0.739	42 (77.8)	47 (87.0)	0.132
Ventricular fibrillation	30 (44.8)	36 (53.7)	0.109	27 (49.1)	24 (43.6)	0.257	33 (61.1)	36 (66.7)	0.257
**Waveform abnormalities**									
Left anterior fascicular block	50 (74.6)	49 (73.1)	0.808	29 (52.7)	24 (43.6)	0.166	39 (72.2)	42 (77.8)	0.257
Left bundle branch block	58 (86.6)	56 (83.6)	0.317	38 (69.1)	38 (69.1)	1	50 (92.6)	49 (90.7)	0.564
Right bundle branch block	55 (82.1)	55 (82.1)	1	42 (76.4)	42 (76.4)	1	48 (88.9)	49 (90.7)	0.655
Wolff-Parkinson-White pattern	49 (73.1)	59 (88.1)	0.012	37 (67.3)	47 (85.5)	0.004	47 (87.0)	52 (96.3)	0.059
Left atrial enlargement	52 (77.6)	51 (76.1)	0.763	38 (69.1)	41 (74.5)	0.439	46 (85.2)	45 (83.3)	0.739
Right atrial enlargement	54 (80.6)	57 (85.1)	0.179	43 (78.2)	45 (81.8)	0.527	47 (87.0)	49 (90.7)	0.414
Left ventricular hypertrophy	46 (68.7)	58 (86.6)	0.003	44 (80.0)	49 (89.1)	0.059	49 (90.7)	51 (94.4)	0.414
Right ventricular hypertrophy	47 (70.1)	63 (94.0)	< 0.001	31 (56.4)	48 (87.3)	< 0.001	44 (81.5)	51 (94.4)	0.020
Anterior STEMI	62 (92.5)	63 (94.0)	0.564	46 (83.6)	48 (87.3)	0.480	52 (96.3)	51 (94.4)	0.564
Inferior STEMI	61 (91.0)	58 (86.6)	0.180	49 (89.1)	48 (87.3)	0.564	44 (81.5)	48 (88.9)	0.046
Pericarditis	30 (44.8)	57 (85.1)	< 0.001	20 (36.4)	40 (72.7)	< 0.001	37 (68.5)	51 (94.4)	0.001
Hyperkalaemia	39 (58.2)	55 (82.1)	< 0.001	18 (32.7)	34 (61.8)	< 0.001	29 (53.7)	45 (83.3)	< 0.001
Prolonged QT interval	54 (80.6)	57 (85.1)	0.405	38 (69.1)	43 (78.2)	0.132	43 (79.6)	48 (88.9)	0.096

Values are N (%). *AV* atrioventricular, *STEMI* ST-segment elevation myocardial infarction

### Impact of prior real life ECG exposure on diagnostic accuracy

**Fig. 5:**
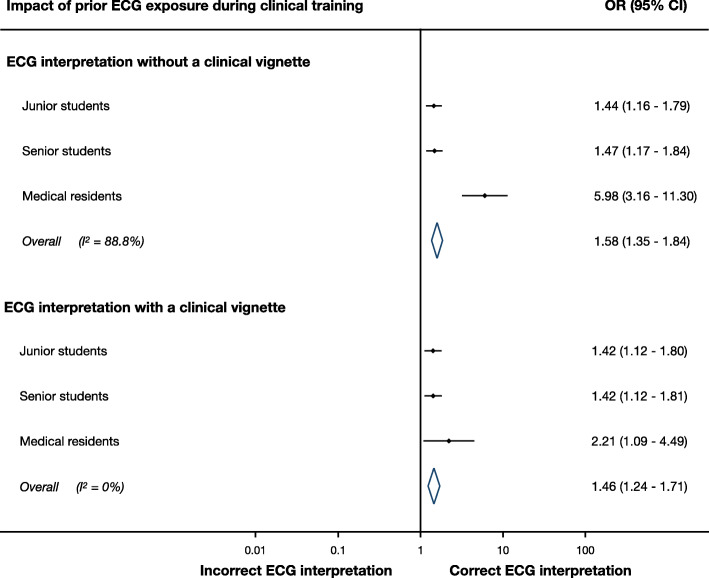
Association between prior ECG exposure during clinical training and ECG diagnostic accuracy, when participants were provided with a case vignette or not

All participants were more likely to make a correct ECG diagnosis if they reported having seen the condition during prior clinical training (Fig. 5). This was true for all groups, whether they were provided with a case vignette (OR 1.46, 95% CI 1.24–1.71) or not (OR 1.58, 95% CI 1.35–1.84). The largest impact was amongst residents, who were almost six times more likely to make a correct diagnosis when they were given an ECG of a condition that they reported to have seen during prior clinical training. The striking feature of this forest plot is that residents’ ECG diagnostic accuracy was not greatly benefited by the provision of a clinical vignette, and that prior exposure to the given ECGs was a more significant influence on ECG diagnostic accuracy.

## Discussion

In this study, we set out to determine whether clinically contextualised ECG interpretation, using case vignettes, improved medical students and residents’ ECG diagnostic accuracy. We found that trainees benefited only marginally from these case vignettes in improving their ECG diagnostic accuracy. However, students and residents were more likely to make the correct ECG diagnosis if they had seen the condition during prior clinical training. In light of this finding, it was of concern that, during clinical clerkships, trainees were not exposed to all the ECGs they were expected to be able to interpret by graduation.

In the classroom, ECGs are often taught without clinical context. In a recent systematic review on computer-assisted ECG training, it was found that only four of thirteen studies used clinical scenarios as part of their training [45]. However, in real life, ECGs are performed in settings where the ECG interpreter is likely to know the clinical presentation. Indeed, we found that students and residents were more accurate at diagnosing RVH, pericarditis and hyperkalaemia, when they knew the clinical context. Of course, in these cases, it is possible that the participant could predict the correct answer from the history alone [43, 46]. However, these ECG diagnoses can be very challenging without knowing the clinical context, especially for the novice ECG interpreter. Knowing the demographic details and risk factor profile of a patient will help to make the differentiation between different conditions causing ST-segment elevation, e.g. pericarditis and myocardial infarction, as opposed to only looking at the ECGs. Similarly, the clinical context is important in the analysis of bradycardias, as hyperkalaemia can easily be mistaken for third degree AV block on an ECG. The distinction is of paramount importance, as the management is very different.

However, one should not consider the impact of case vignettes on the interpretation of single ECG diagnoses. In this study, we tested the impact of case vignettes on the interpretation of ECGs on a wide array of arrhythmias and waveform abnormalities. We found that the largest value in improved diagnostic accuracy was for ECGs showing abnormal waveforms, as has been described by Hatala et al. [42] Our study further supports their findings, in that medical trainees with different levels of expertise all benefited from being provided with a clinical scenario when interpreting ECGs with abnormal waveforms. Our study also supports the study by Grum et al., that showed that clinical scenarios had no significant impact on junior medical students’ interpretation of atrial fibrillation and supraventricular tachycardia [41]. In addition to the latter, our study also included AV blocks, atrial flutter and ventricular tachycardia. We found that junior medical students (with little clinical experience) did not benefit from case vignettes when interpreting these arrhythmias. This could potentially be explained by their lack of exposure to patients presenting with rhythm disturbances during clinical clerkships in general medical wards. This is a cause for major concern, as there is little formal ECG training after graduation [30].

Medical training has traditionally relied on experiential learning, i.e., the acquisition of knowledge and skills from clinical exposure [47]. Indeed, there is a close relationship between the accrual of clinical experience and increased competence [48]. As has been shown for the interpretation of chest radiographs [49, 50], we found that ECG diagnostic accuracy increased with more advanced levels of training. Our study results were also consistent with the literature reporting that diagnostic accuracy is positively influenced by prior exposure to examples of similar conditions [51]. Moreover, increased exposure and repeated practice are known to be associated with better ECG diagnostic accuracy [52, 53]. We therefore propose that ECG exposure should be maximised during clinical clerkships, to ensure that undergraduate and postgraduate trainees become familiar with the conditions specified and recommended by undergraduate [30–32] and postgraduate ECG curricula [33–38].

Merely being present on ward rounds or in the clinic or emergency unit, does not result in the acquisition of ECG competence. Instead, students and residents should be actively encouraged to analyse and interpret ECGs during their clinical training. This largely self-directed learning pursuit may assist in gaining more experience, as well as contextualising ECG learning [54]. ECG learning in the clinical setting should be supported by using mobile learning strategies [55], which may further enhance contextualised learning [56, 57]. In this regard, there is evidence that the use of algorithm-based ECG reference apps may be of greater benefit than unguided exploration of the Internet [58]. However, there is limited exposure during clinical clerkships to conditions that medical students are expected to diagnose [30]. This implies that ECG training should not rely on experiential learning alone for teaching electrocardiography. Instead, ECG training should be supplemented by other formal methods of instruction [45, 59, 60].

### Study limitations

We acknowledge that, according to the hierarchy of study design, randomised control trials (RCT) provide a better level of evidence and pose less risk of bias than cross-sectional studies. However, study design depends on the research question and available resources to conduct a study. Through randomised allocation, RCTs ensure that the cohorts studied have identical baseline characteristics, to assess the effect of an intervention. However, cross-sectional studies provide a snapshot in time, acknowledging different baseline characteristics of the cohorts in the study. For this reason, we purposefully chose a cross-sectional study design, as it allowed us to assess the accuracy of ECG interpretation when participants with different levels of experience, i.e., students and residents, were provided with a case vignette or not at the time of ECG interpretation. As all participants interpreted the same ECGs with and without clinical scenarios, each participant served as their own control in this study.

We recognise that generalisability of our findings is limited to our experience (i.e., undergraduate clinical clerkships and postgraduate residency programme at our institution), and would need to be studied in other contexts to confirm its global relevance. However, it highlights that, unless a medical student spends dedicated time in clinical clerkships such as Cardiology or Emergency Medicine during their undergraduate training, they might not encounter all conditions recommended for ECG training [30]. We also acknowledge that the study could not control for factors likely to influence performance bias, such as additional *ad hoc* ECG teaching, and the additional training of postgraduate trainees.

Our study only tested the impact of case vignettes that were appropriate to the ECGs that were provided. We did not include misleading clinical scenarios, to evaluate if these would impede diagnostic accuracy. Although only tested on limited ECGs, it has been shown before that misleading case vignettes are detrimental to ECG diagnostic accuracy [42].

## Conclusion

The main message of this study is that clinical exposure to patients with ECG abnormalities during clinical clerkships plays an important role in learning Electrocardiography. Contextualised ECG interpretation using case vignettes has a limited impact on ECG diagnostic accuracy if trainees have not had prior exposure to these conditions during real patient encounters. However, since exposure to ECGs during clinical training is opportunistic and haphazard, ECG training cannot rely solely on contextual learning during clinical clerkship training. The mandate to supplement contextual learning with structured learning activities in the workplace, including mobile learning, is clear. The evidence to support these learning strategies is emerging in the literature.

## Supplementary Information

**Additional file 1.**

## Data Availability

The datasets used and/or analysed during the current study, are available in the “*Clinically contextualised ECG interpretation”* repository, which could be accessed at 10.25375/uct.14297708.v1. The ECGs contained in the supplementary material are the property of the University of Cape Town, and when used, these should be referenced as such.

## Abbreviations

AF: Atrial fibrillation
AV: Atrioventricular
AVNRT: AV nodal re-entrant tachycardia
CI: confidence interval
ECG: Electrocardiogram
LAFB: Left anterior fascicular block
LBBB: Left bundle branch block
LVH: Left ventricular hypertrophy
MCQ: Multiple-choice question
OR: Odds ratio
RBBB: Right bundle branch block
RVH: Right ventricular hypertrophy
STEMI: ST-segment elevation myocardial infarction
UCT: University of Cape Town
VF: Ventricular fibrillation
VT: Ventricular tachycardia
WPW: Wolff-Parkinson-White
