# Supplementary material for: Clinically contextualised ECG interpretation: the impact of prior clinical exposure and case vignettes on ECG diagnostic accuracy
Source: BMC Med Educ. 2021 Aug 3;21:417. doi: 10.1186/s12909-021-02854-x (PMC8336410; doi:10.1186/s12909-021-02854-x)
Supplement: Supplementary file 1 — Additional file 1. [file 12909_2021_2854_MOESM1_ESM.pdf]

ECGs and multiple-choice questions (MCQs) used for the study
“Clinically contextualised ECG interpretation by medical trainees”

In Test 1, the ECGs were shown without the clinical vignettes.
In Test 2, the ECGs were shown with the clinical vignettes.

The ECGs contained in this document are the property of the University of Cape Town.
If used, they should be referenced as such.

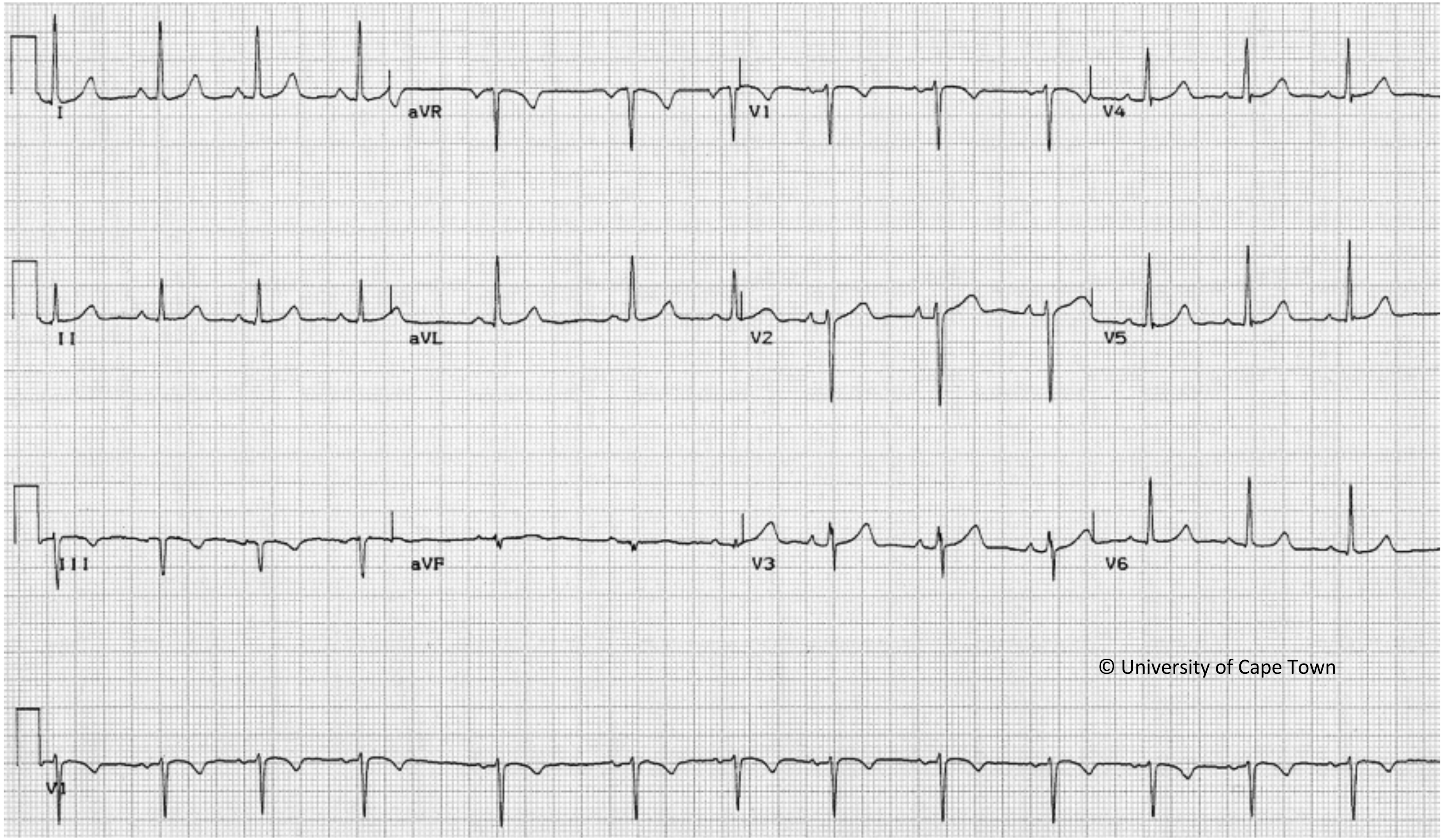

1. **A 31 year old man who is otherwise healthy undergoes a medical examination with ECG for insurance purposes**.<br>How do you describe the rhythm of this ECG?	
	a. Sinus rhythm
x	b. Sinus arrhythmia
	c. Atrial fibrillation
	d. Mobitz type I second degree AV block
	e. I do not know the answer

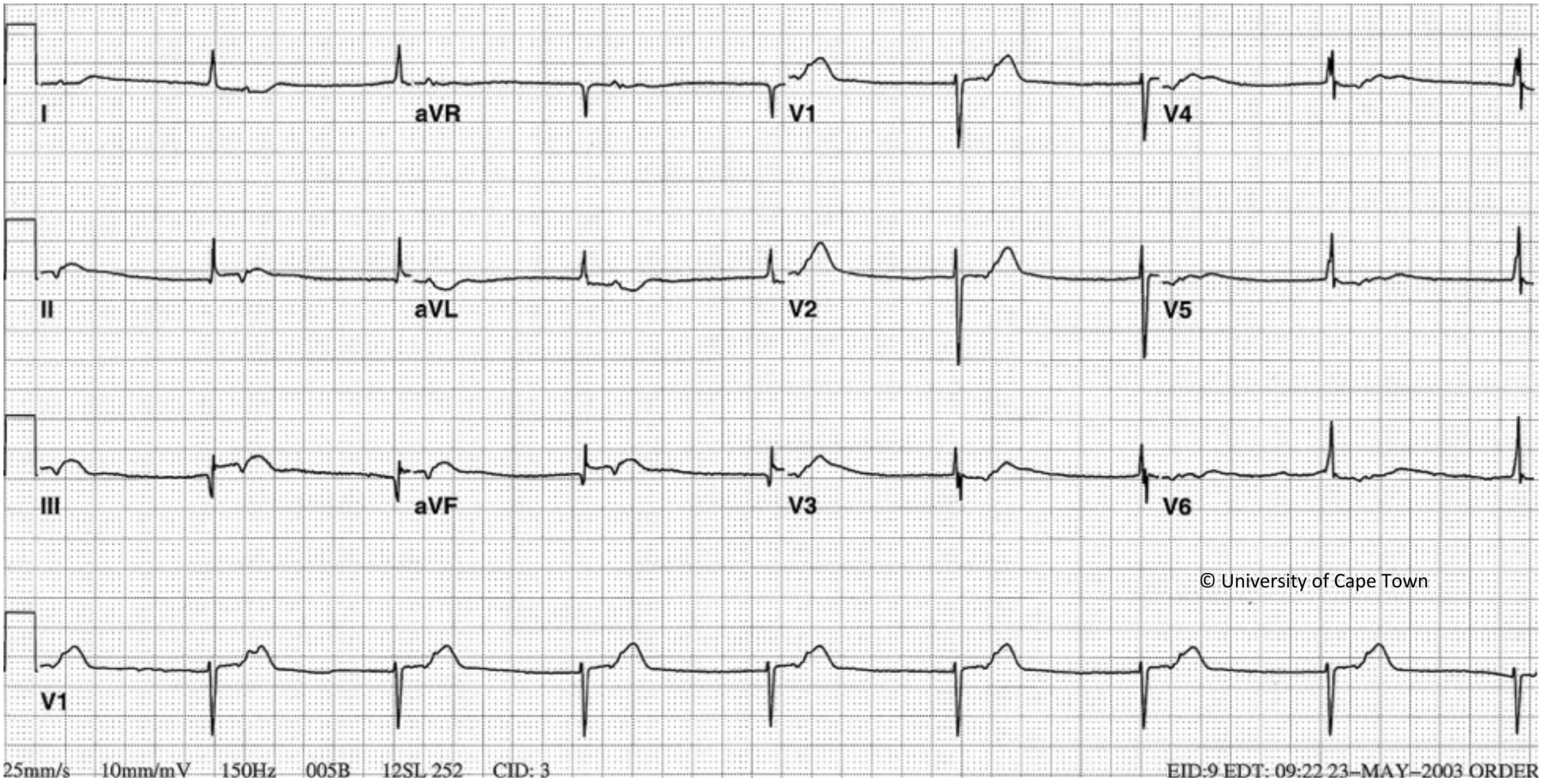

2. **An ambulance arrives with a conscious 60 year old male patient with chest discomfort who collapsed in a shopping mall. His BP is 80/50**.<br>How do you describe the rhythm?	
	a. Normal sinus rhythm
x	b. Sinus arrest with junctional escape
	c. Mobitz type II second degree AV block
	d. Third degree AV block
	e. I do not know the answer

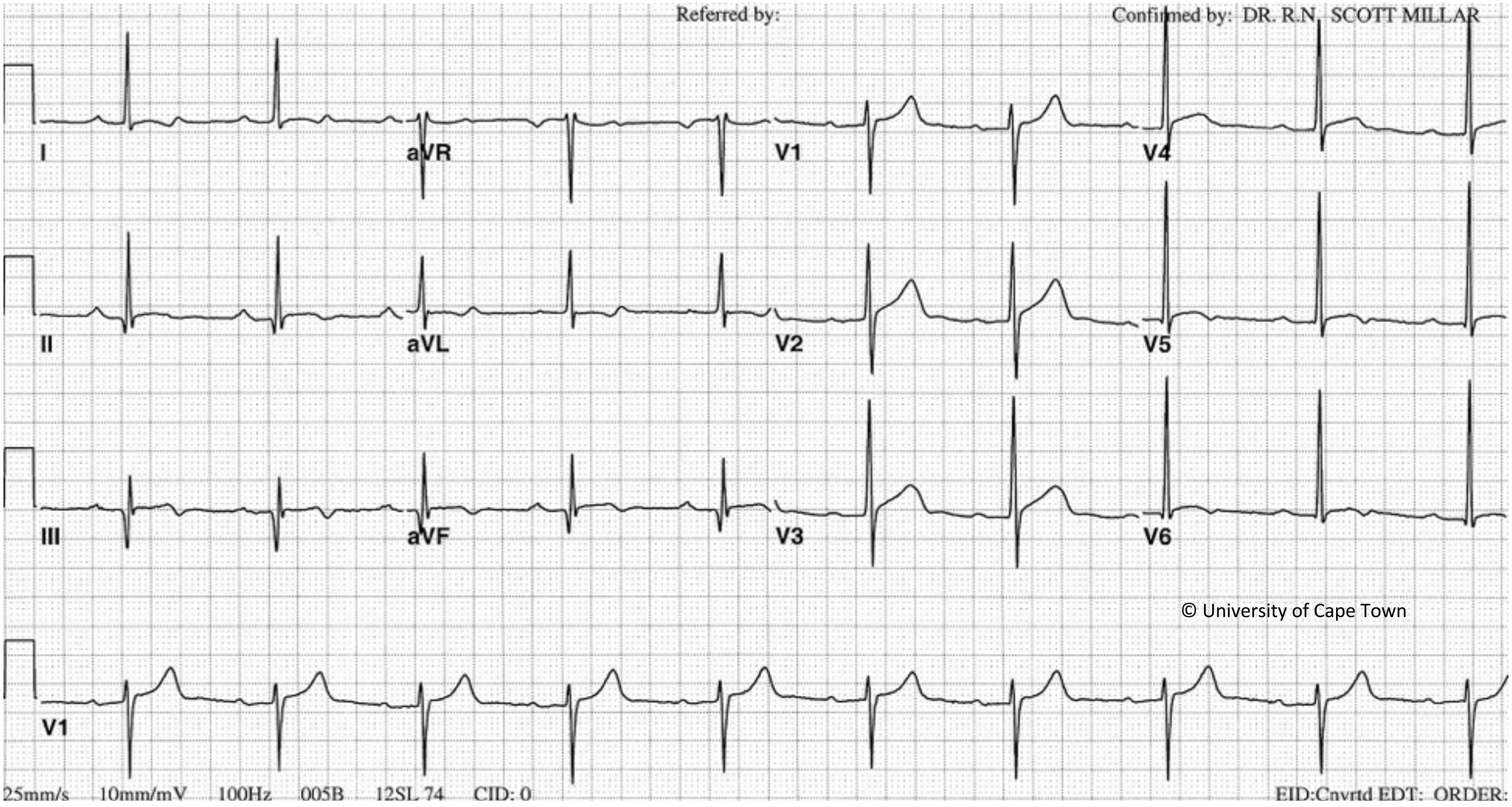

	3. **An ECG is done on a 23 year old female patient with acute rheumatic fever.** What is the abnormality on this ECG?
	a. U waves
	b. 2:1 AV block
x	c. First degree AV block
	d. Mobitz type II second degree AV block
	e. I do not know the answer

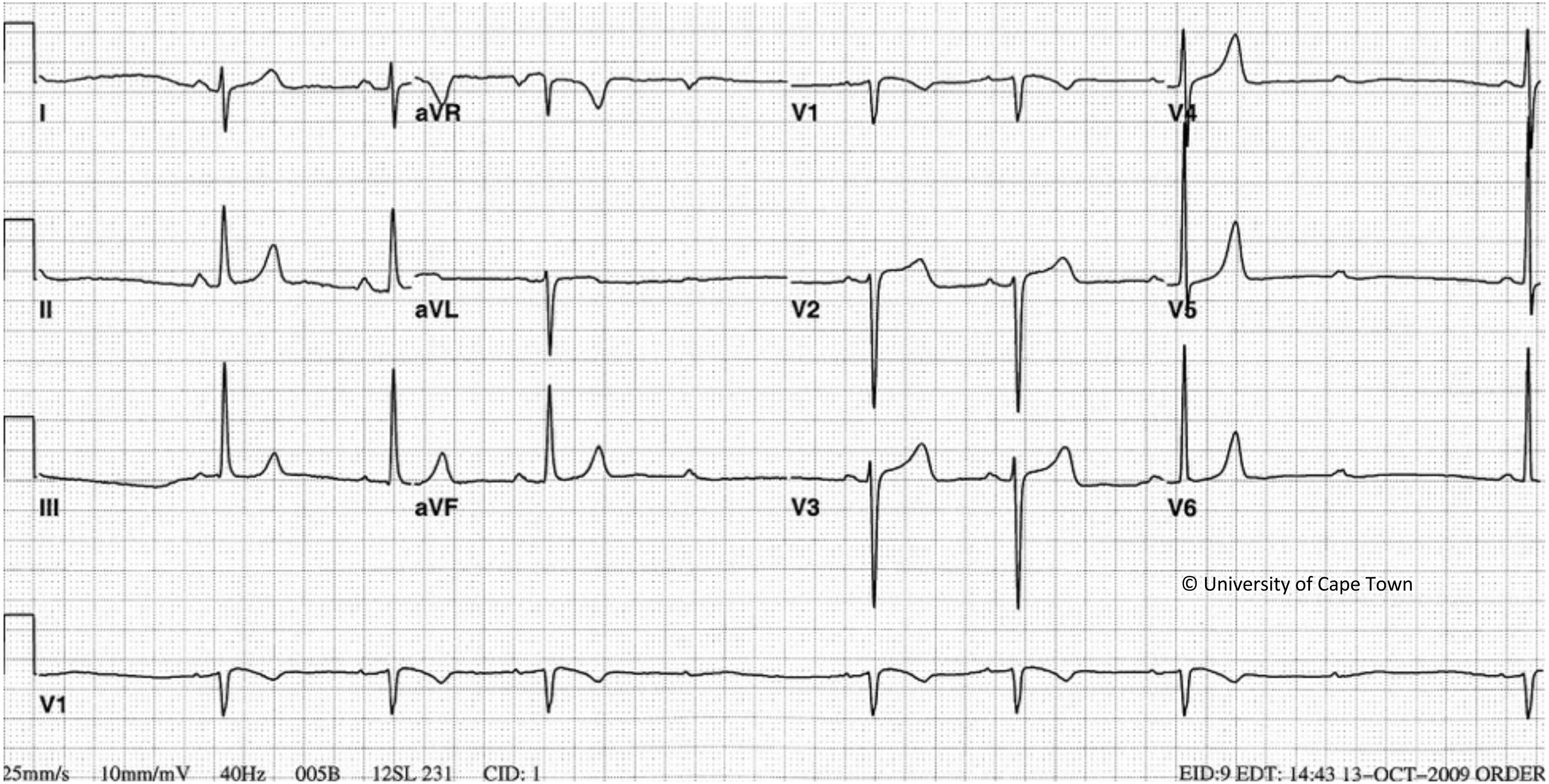

4. **A 72 year old man presents with dizziness to the emergency department. He is stable, but his pulse is irregular. An ECG is done.** What is your diagnosis?	
	a. First degree AV block
x	b. Mobitz type I second degree AV block
	c. Mobitz type II second degree AV block
	d. Third degree AV block
	e. I do not know the answer

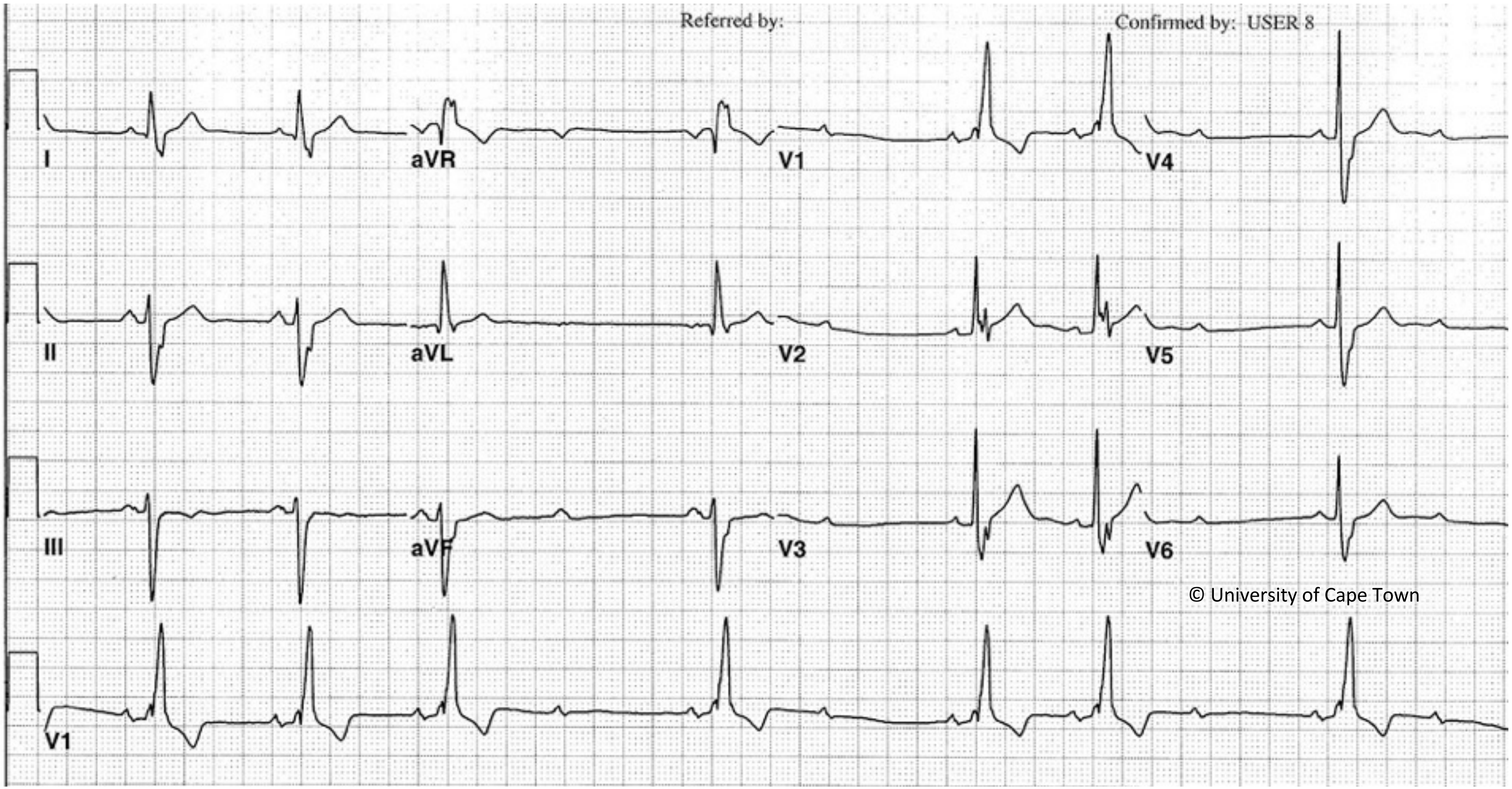

© University of Cape Town

5. **A 62 year old man with recent myocardial infarction presents with dizziness and mild dyspnoea. The blood pressure is 150/90 on arrival.** What is your diagnosis?	
	a. First degree AV block
	b. Mobitz type I second degree AV block
x	c. Mobitz type II second degree AV block
	d. Third degree AV block
	e. I do not know the answer

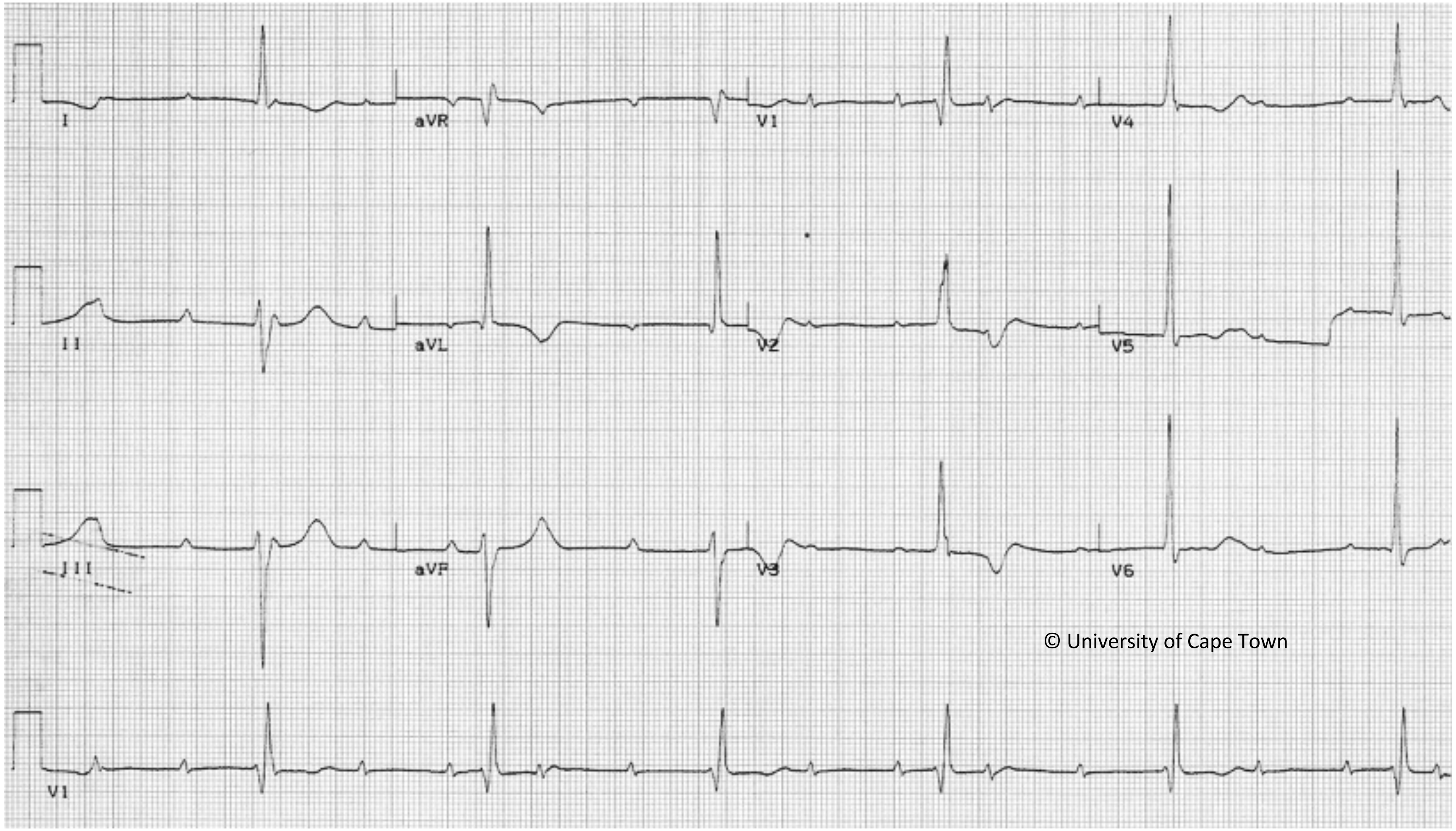

	6. **71 year old man presents with a history of fatigue and syncope. His BP is 183/91. His JVP is slightly raised with canon a waves and on auscultation he has varying intensity of the first heart sound.** What is the diagnosis?
	a. First degree AV block
	b. Mobitz type I second degree AV block
	c. Mobitz type II second degree AV block
x	d. Third degree AV block
	e. I do not know the answer

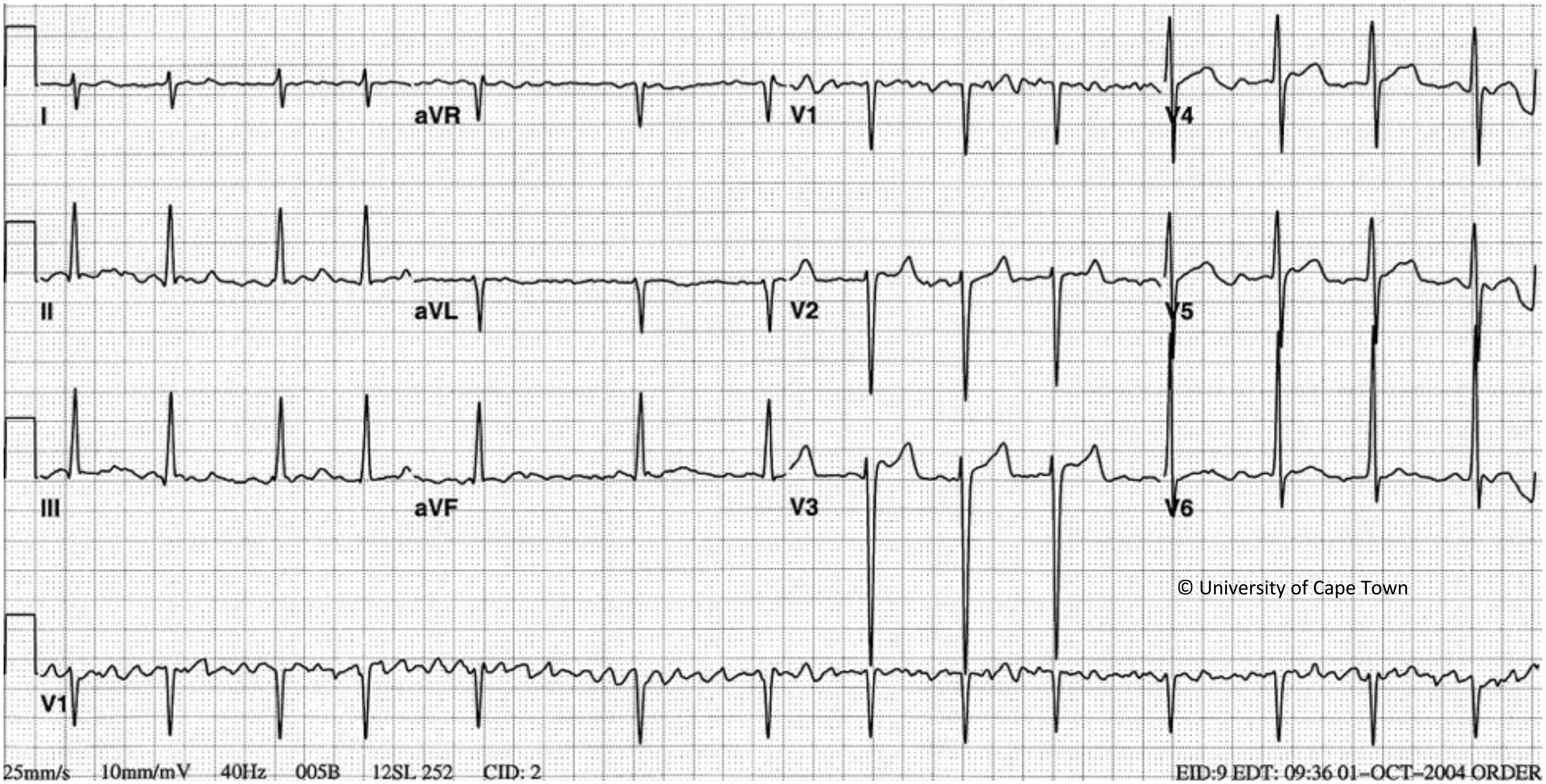

7. **An ECG is done on a 68 year old man with hypertension who presents with a stroke.**<br>What is your diagnosis?	
x	a. Atrial fibrillation
	b. Atrial flutter with variable AV block
	c. Mobitz type I AV block
	d. Mobitz type II AV block
	e. I do not know the answer

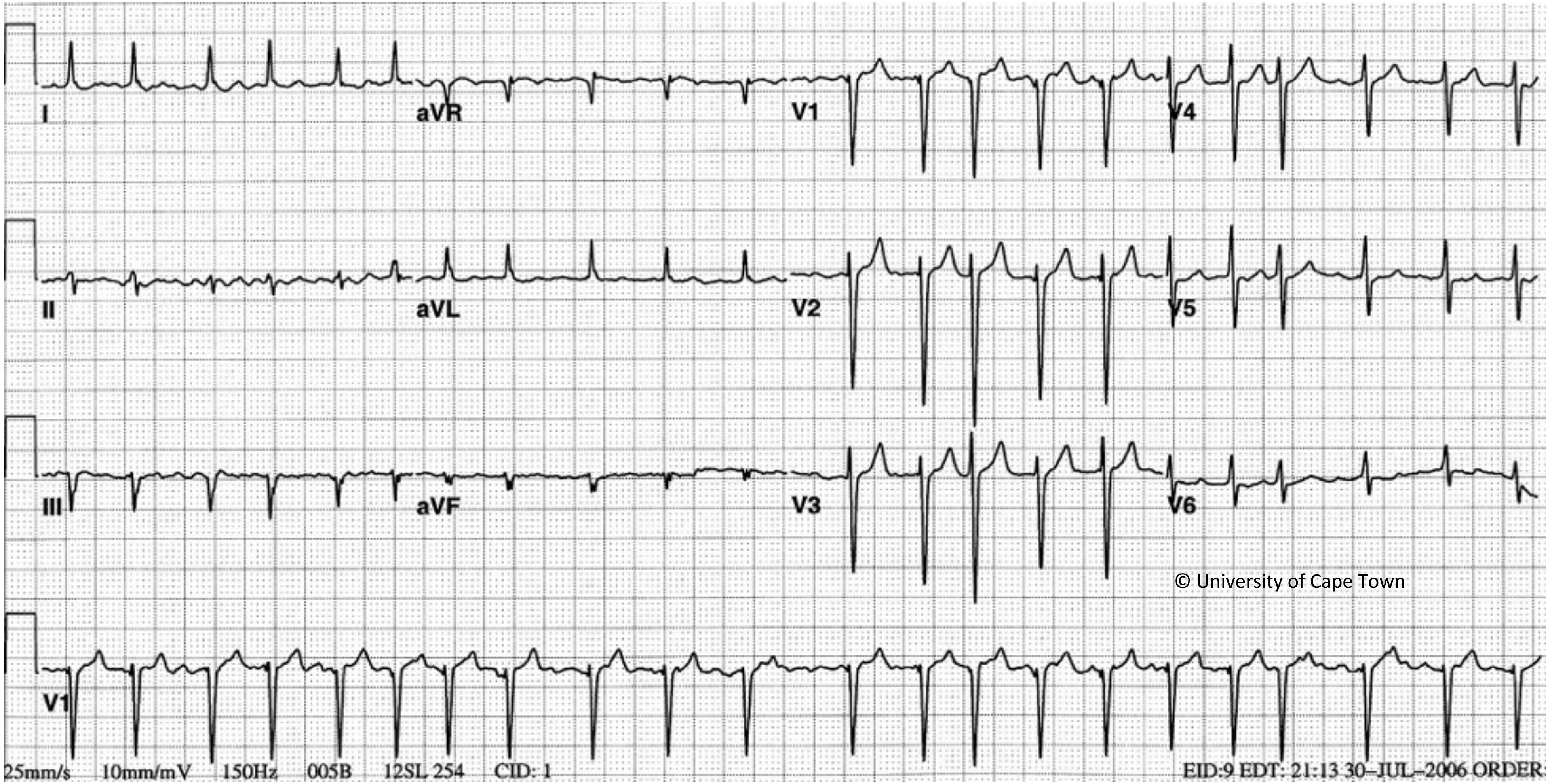

© University of Cape Town

8. **A 73 year old woman, on antihypertensives and oral anticoagulation, is admitted to hospital for treatment of her pneumonia.** What do you diagnose on her ECG?	
	a. Sinus tachycardia
	b. Multifocal atrial tachycardia
x	c. Atrial fibrillation with uncontrolled rate
	d. Atrial flutter with variable AV block
	e. I do not know the answer

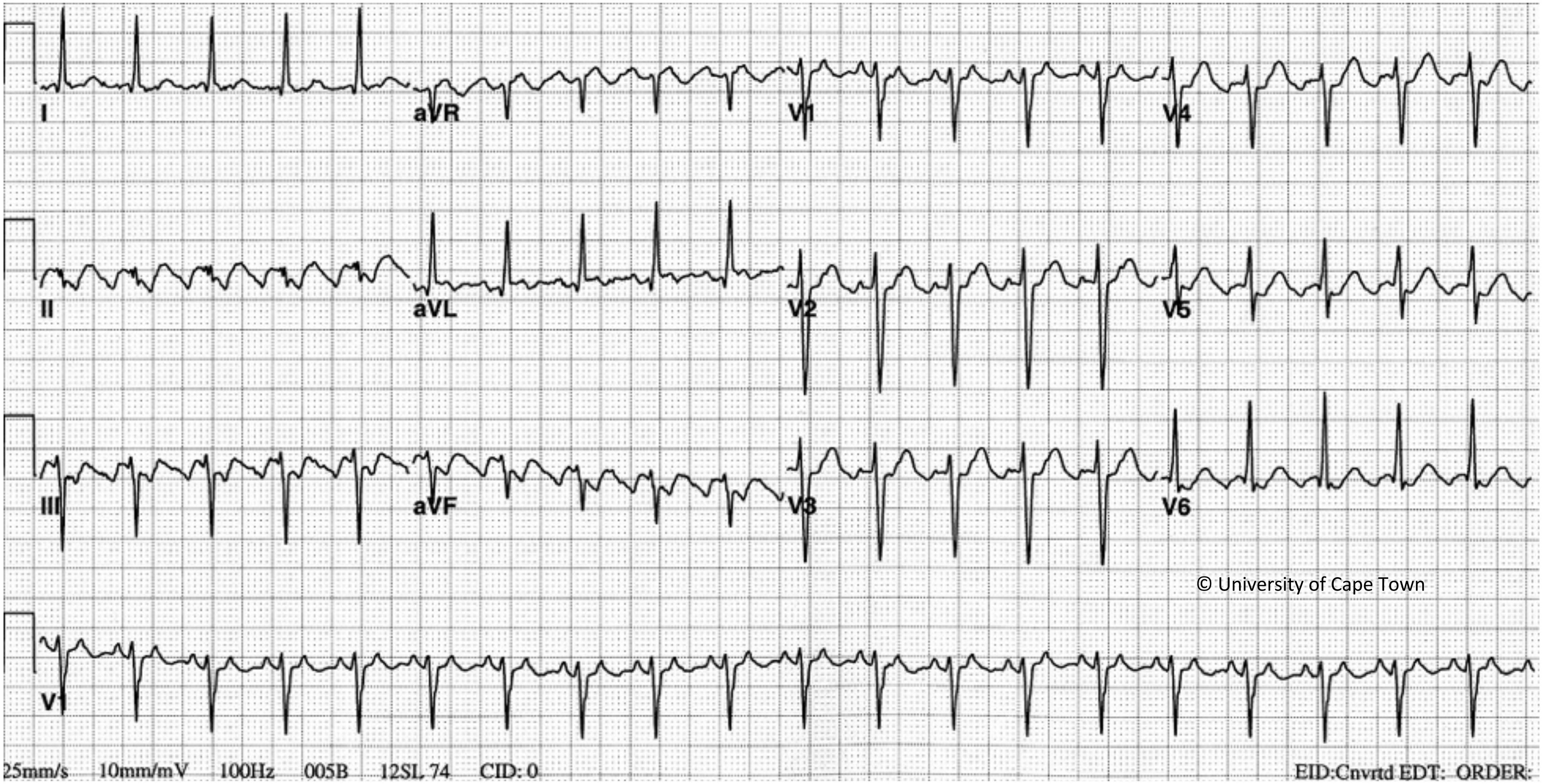

© University of Cape Town

9. **A 55 year old male with previous mitral valve replacement presents with palpitations and mild shortness of breath.** What is your diagnosis?	
	a. Atrial fibrillation
x	b. Atrial flutter with 2:1 AV block
	c. Atrial tachycardia
	d. AV nodal re-entrant tachycardia
	e. I do not know the answer

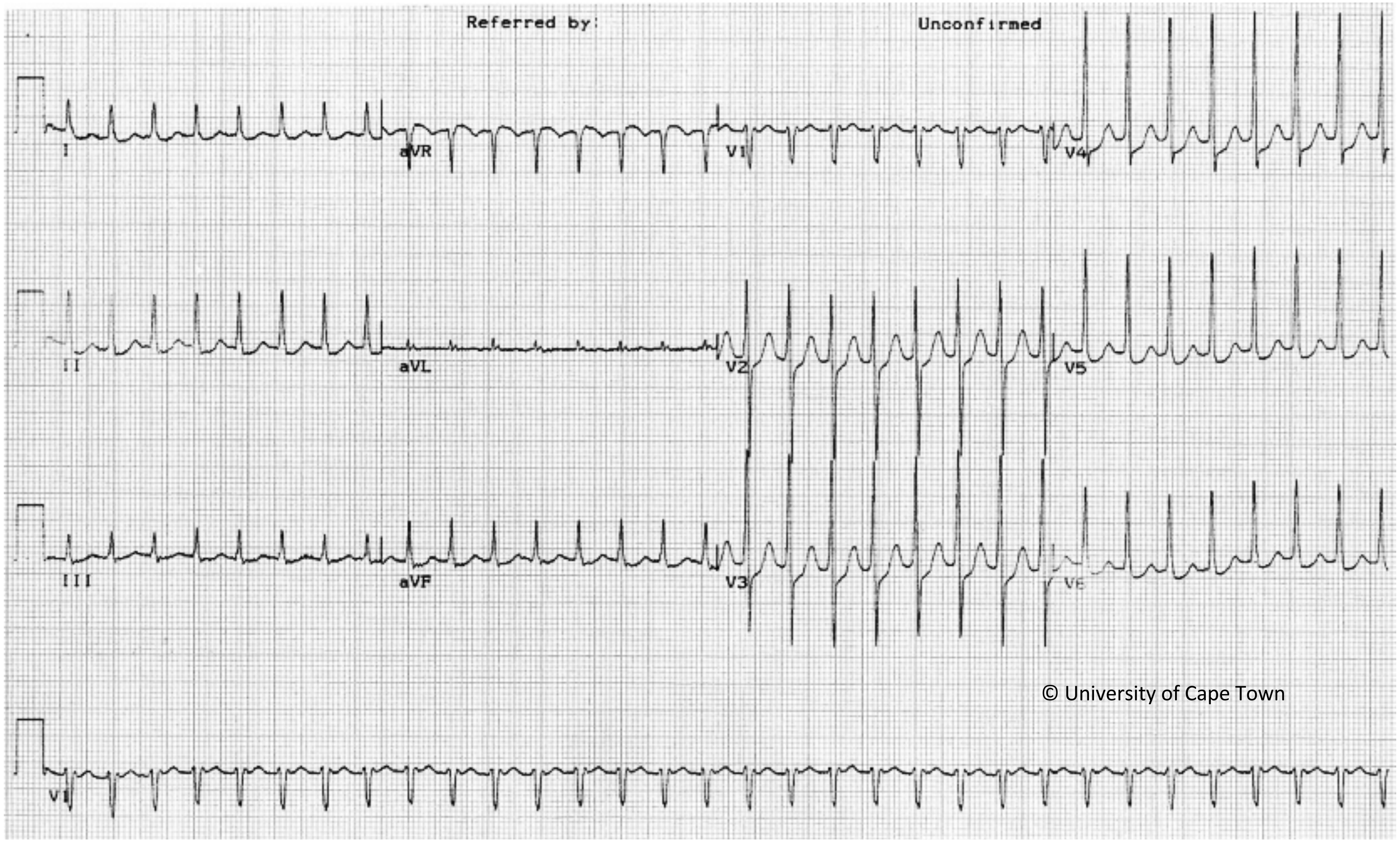

© University of Cape Town

10. **A 47 year old female who is otherwise well presents with a history of abrupt onset of palpitations, terminated by vagal manoeuvres.** What is your diagnosis?	
	a. Atrial fibrillation with uncontrolled rate
	b. Sinus tachycardia
	c. Multifocal atrial tachycardia
x	d. AV nodal re-entrant tachycardia (AVNRT)
	e. I do not know the answer

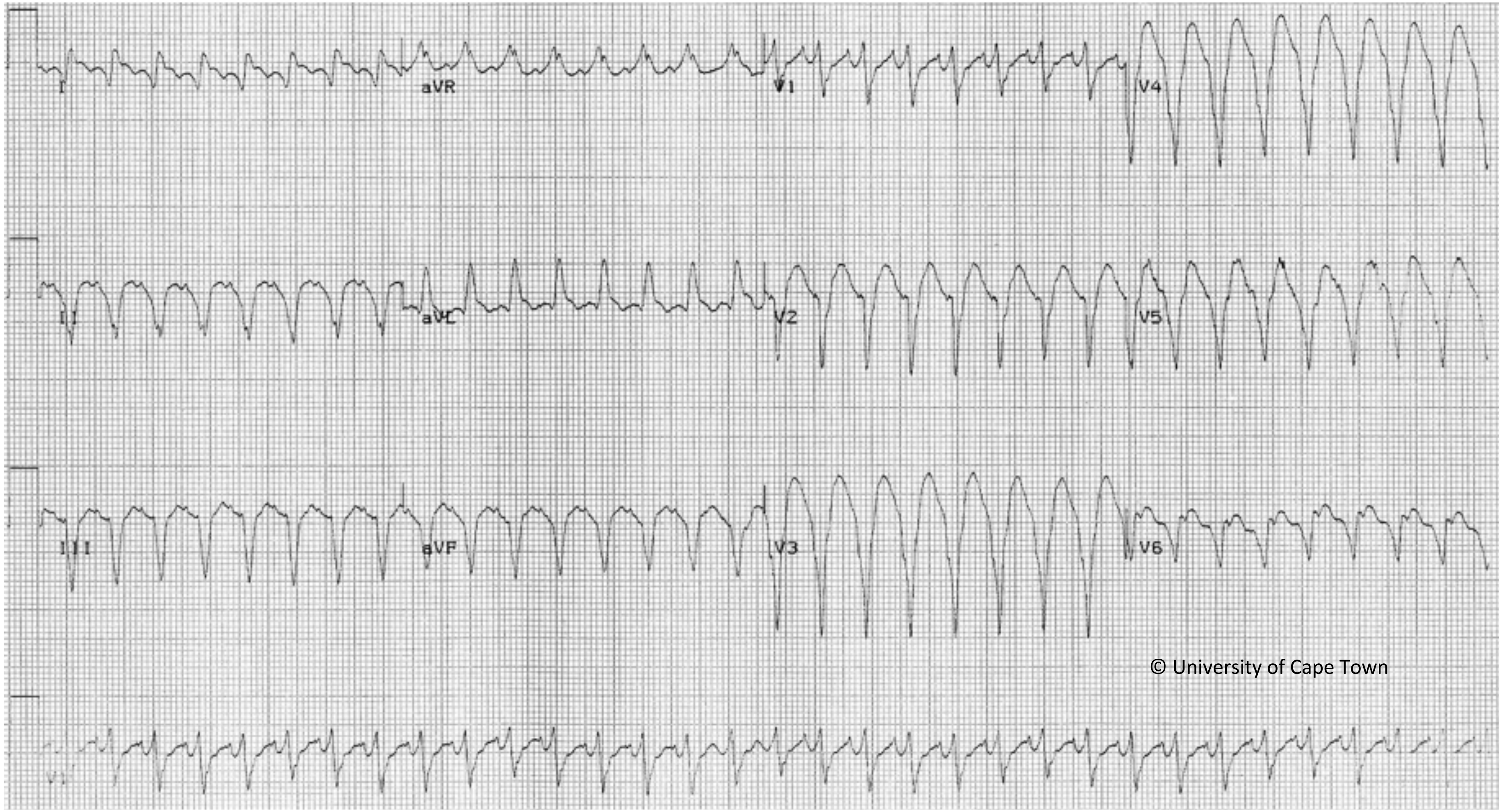

11. **57 year old man with a previous myocardial infarction presents with chest pain, palpitations and syncope, with a BP of 70/40.** What is your diagnosis?	
	a. Supraventricular tachycardia (SVT) with left bundle branch block (LBBB)
	b. Antidromic AV re-entrant tachycardia (AVNRT)
x	c. Monomorphic ventricular tachycardia (VT)
	d. Paced rhythm
	e. I do not know the answer

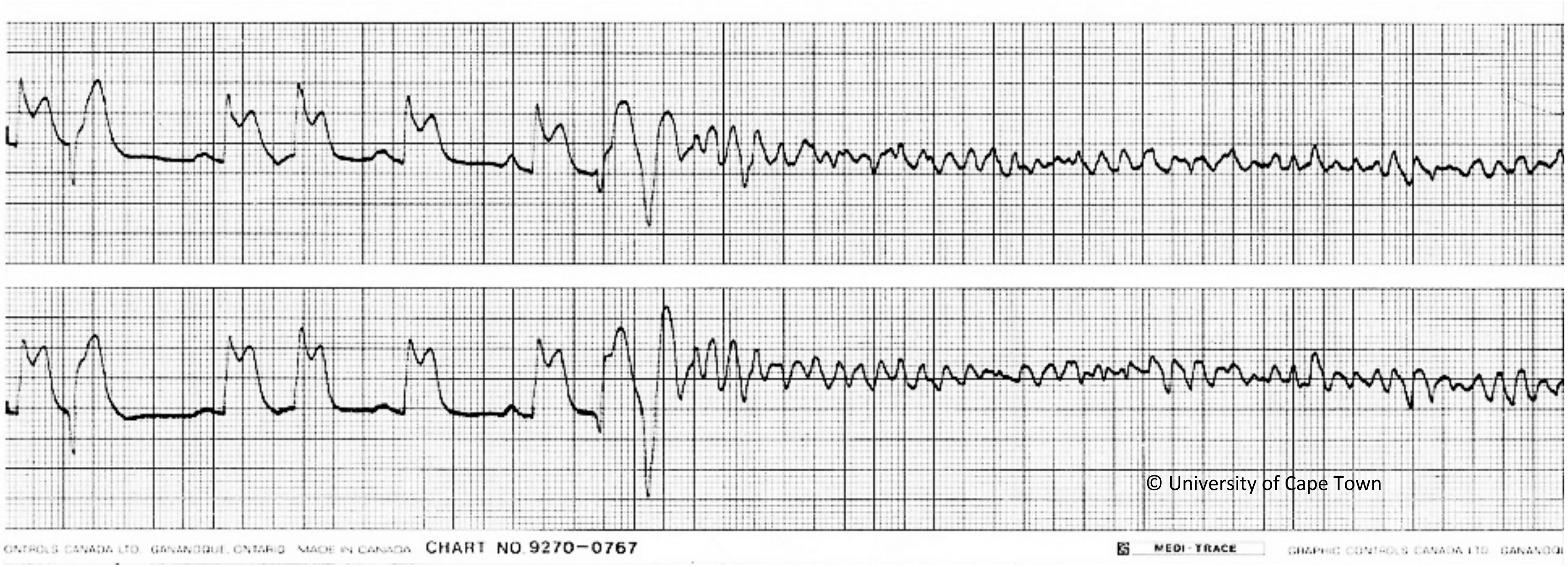

12. **A patient with chest pain loses consciousness and no pulse can be palpated. Resuscitation is started.**<br>How would you best describe the rhythm strip?	
x	a. Ventricular fibrillation (VF)
	b. Torsades de pointes (TdP)
	c. Monomorphic ventricular tachycardia (VT)
	d. Pulseless electrical activity (PEA)
	e. I do not know the answer

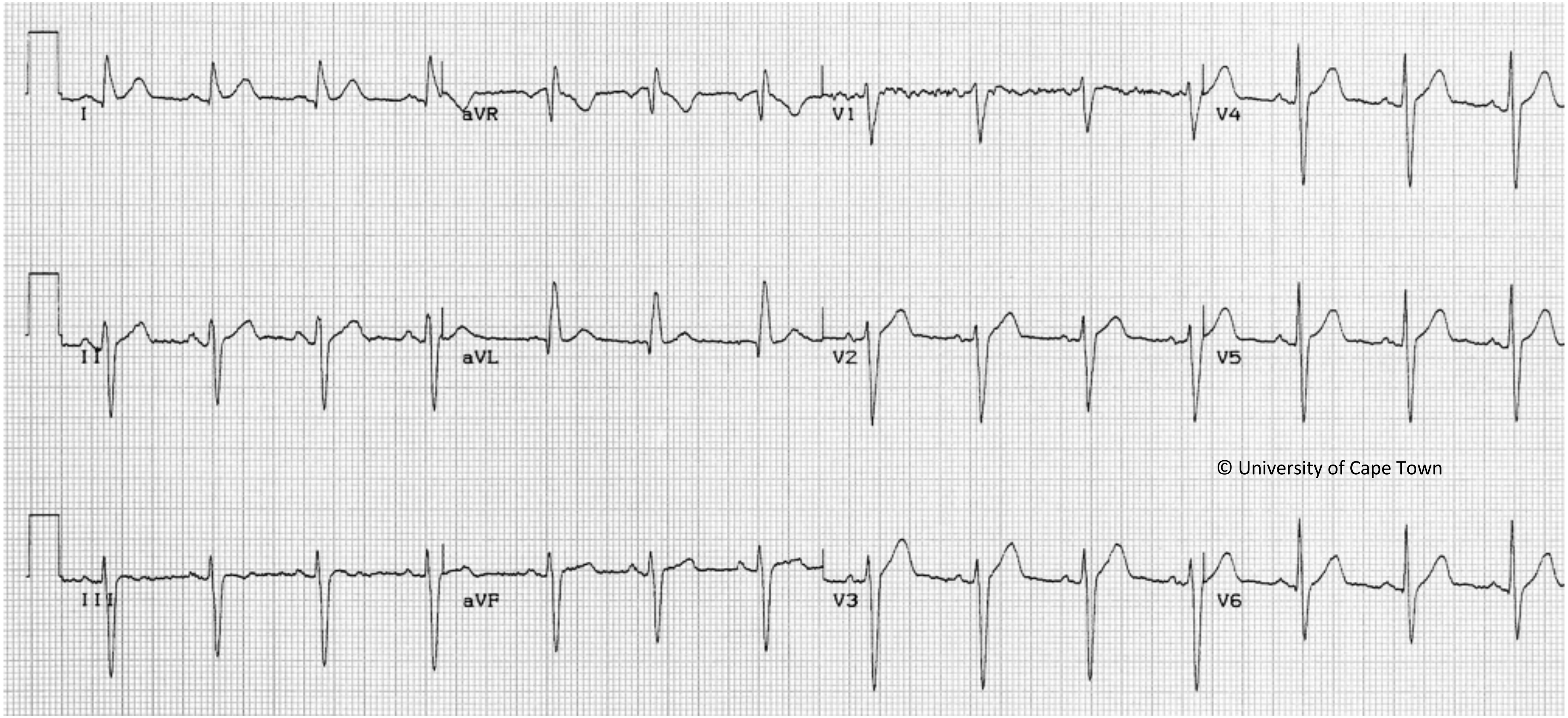

© University of Cape Town

13. **An ECG is performed on a 65 year old male patient with hypertension who is admitted for elective surgery. He is otherwise well. You are called to interpret the ECG.**<br>What is the abnormality on this ECG?	
	a. This is a normal ECG
	b. Right ventricular hypertrophy (RVH)
x	c. Left anterior fascicular block (LAFB)
	d. 2:1 AV block
	e. I do not know the answer

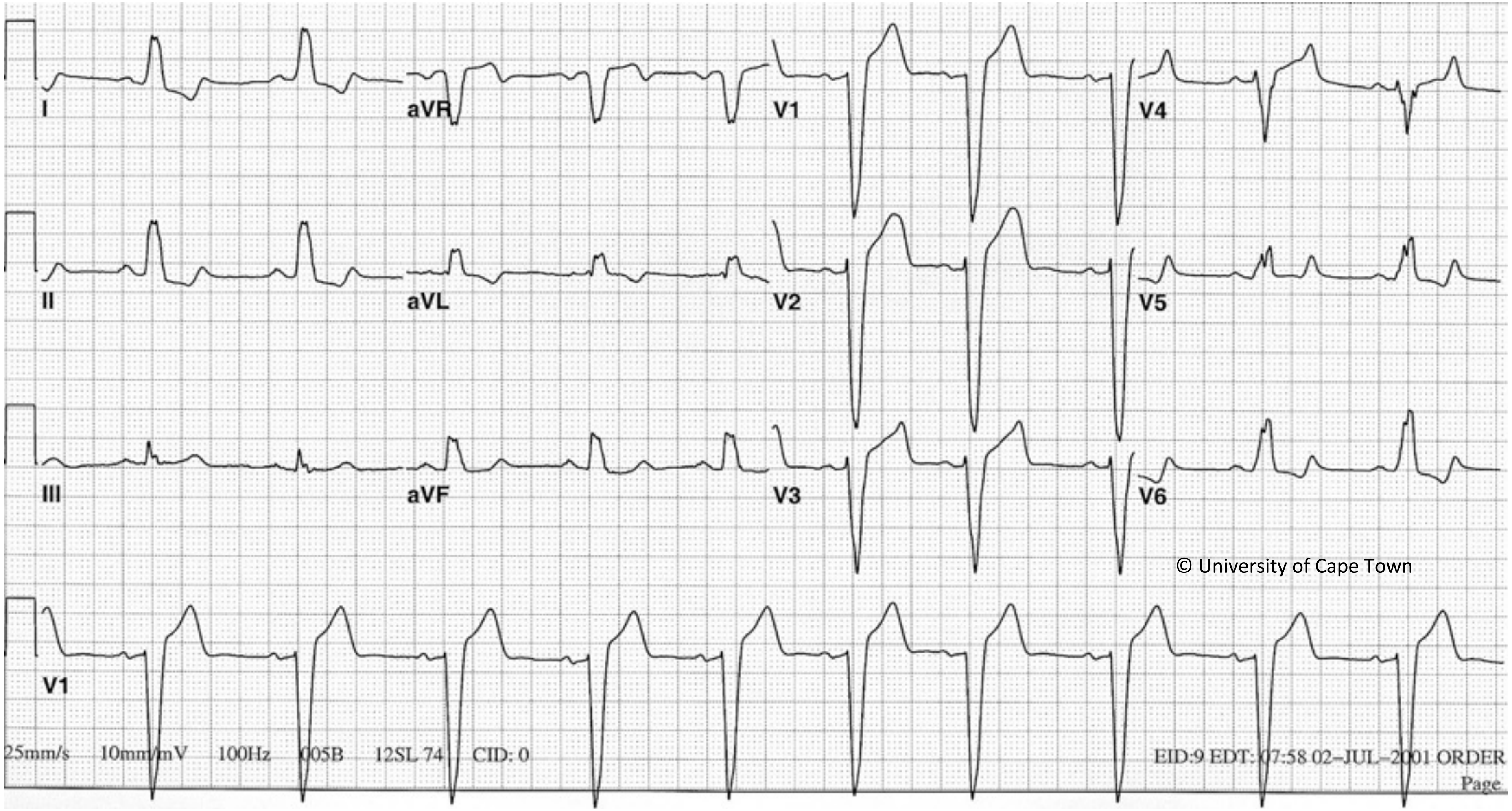

14. **An ECG is done on a 74 year old woman with hypertension and coronary artery disease, admitted for a total knee replacement**.<br>What is the abnormality on this ECG?	
	a. Right bundle branch block (RBBB)
x	b. Left bundle branch block (LBBB)
	c. Wolff-Parkinson-White (WPW) syndrome
	d. Left posterior fascicular block (LPFB)
	e. I do not know the answer

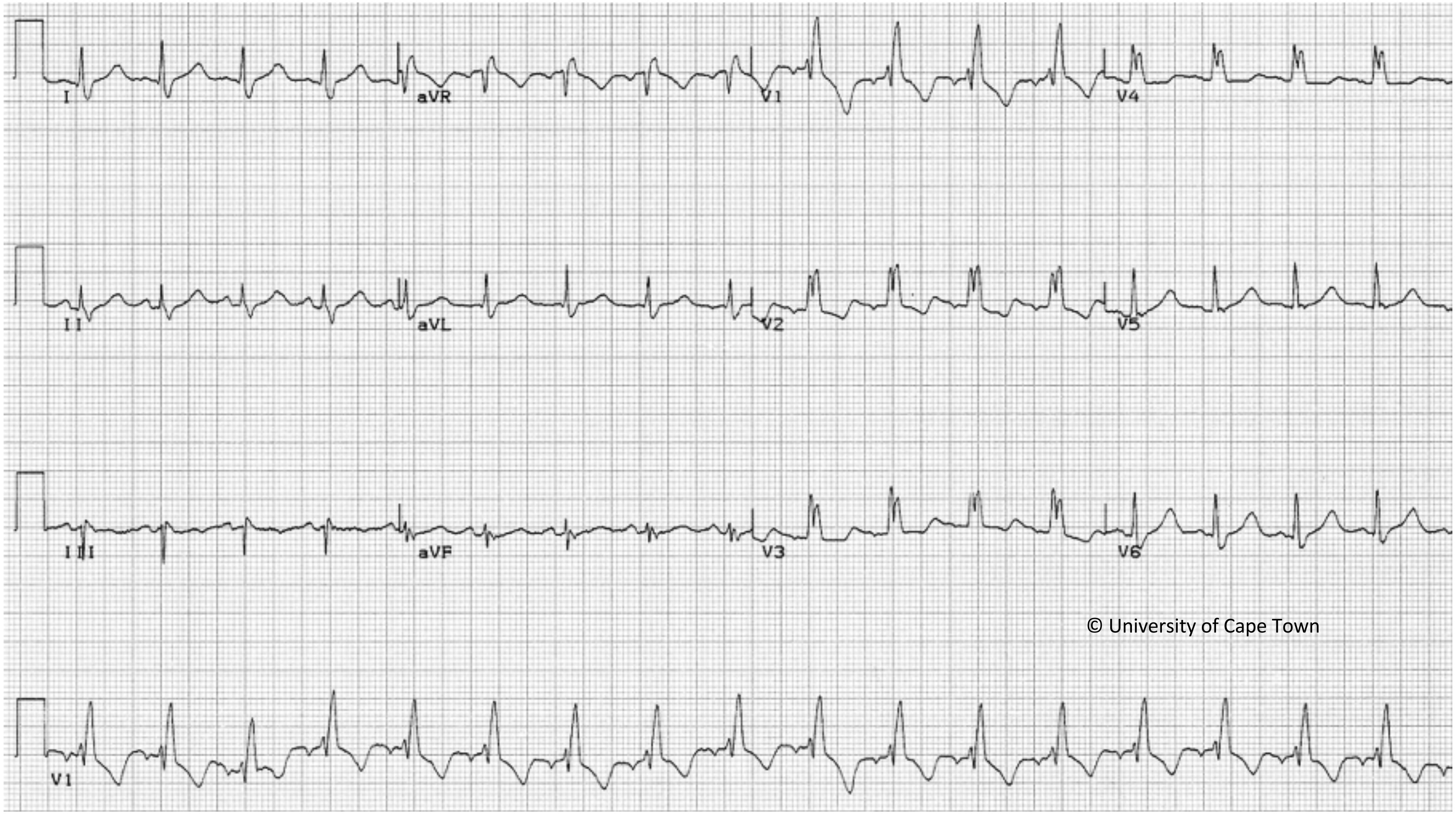

15. **An ECG is done on a 47 year old woman who takes lifelong warfarin for recurrent pulmonary emboli.** What is the abnormality on this ECG?	
x	a. Right bundle branch block (RBBB)
	b. Left bundle branch block (LBBB)
	c. Left anterior fascicular block (LAFB)
	d. Left posterior fascicular block (LPFB)
	e. I do not know the answer

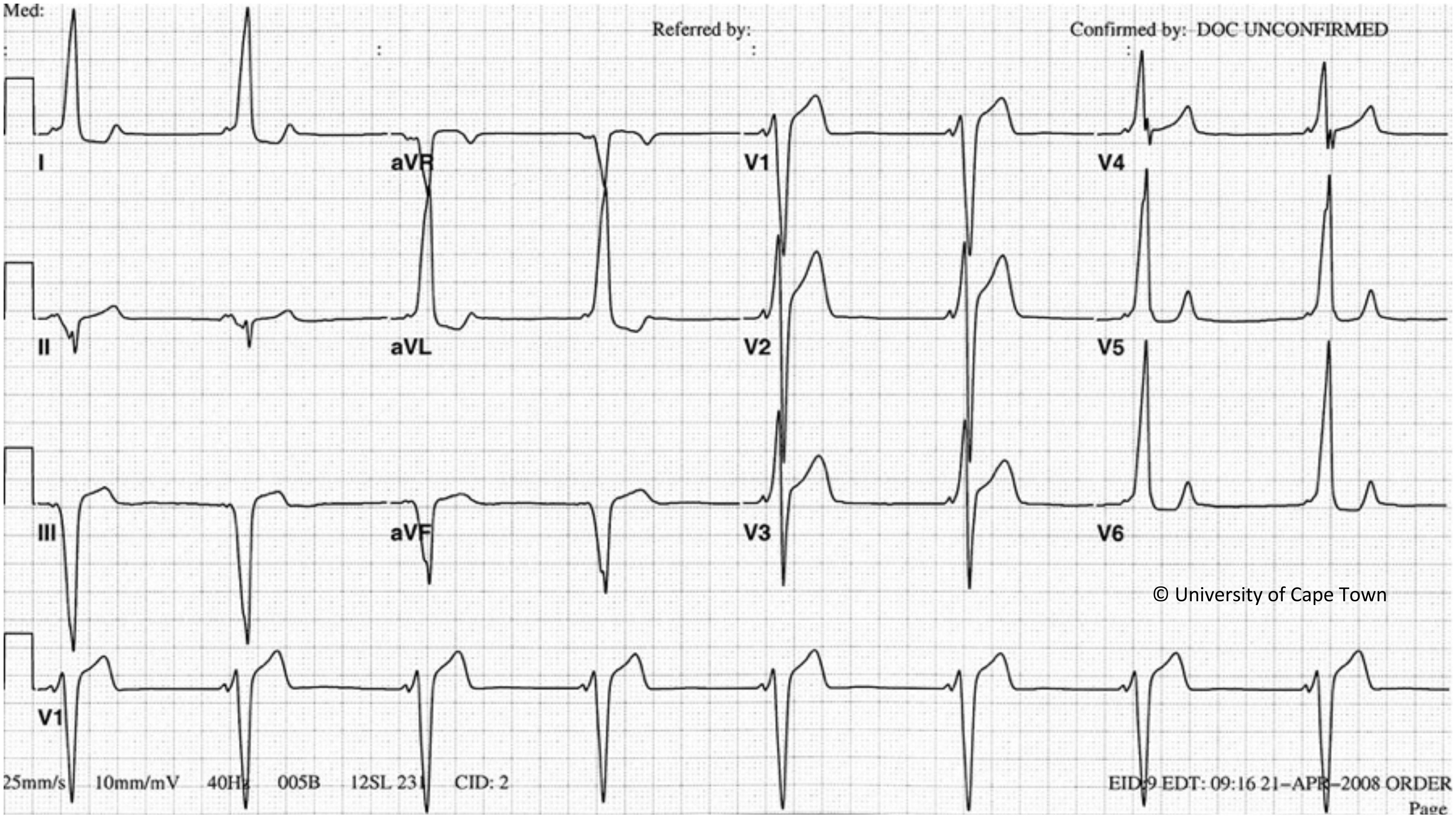

16. **A 16 year old boy presents with occasional rapid palpitations and shortness of breath, during which time he feels dizzy.**
What diagnosis can you make from this ECG?

	a. Hyperkalaemia
	b. Left bundle branch block (LBBB)
	c. Left ventricular hypertrophy (LVH)
x	d. Wolf-Parkinson-White (WPW) syndrome
	e. I do not know the answer

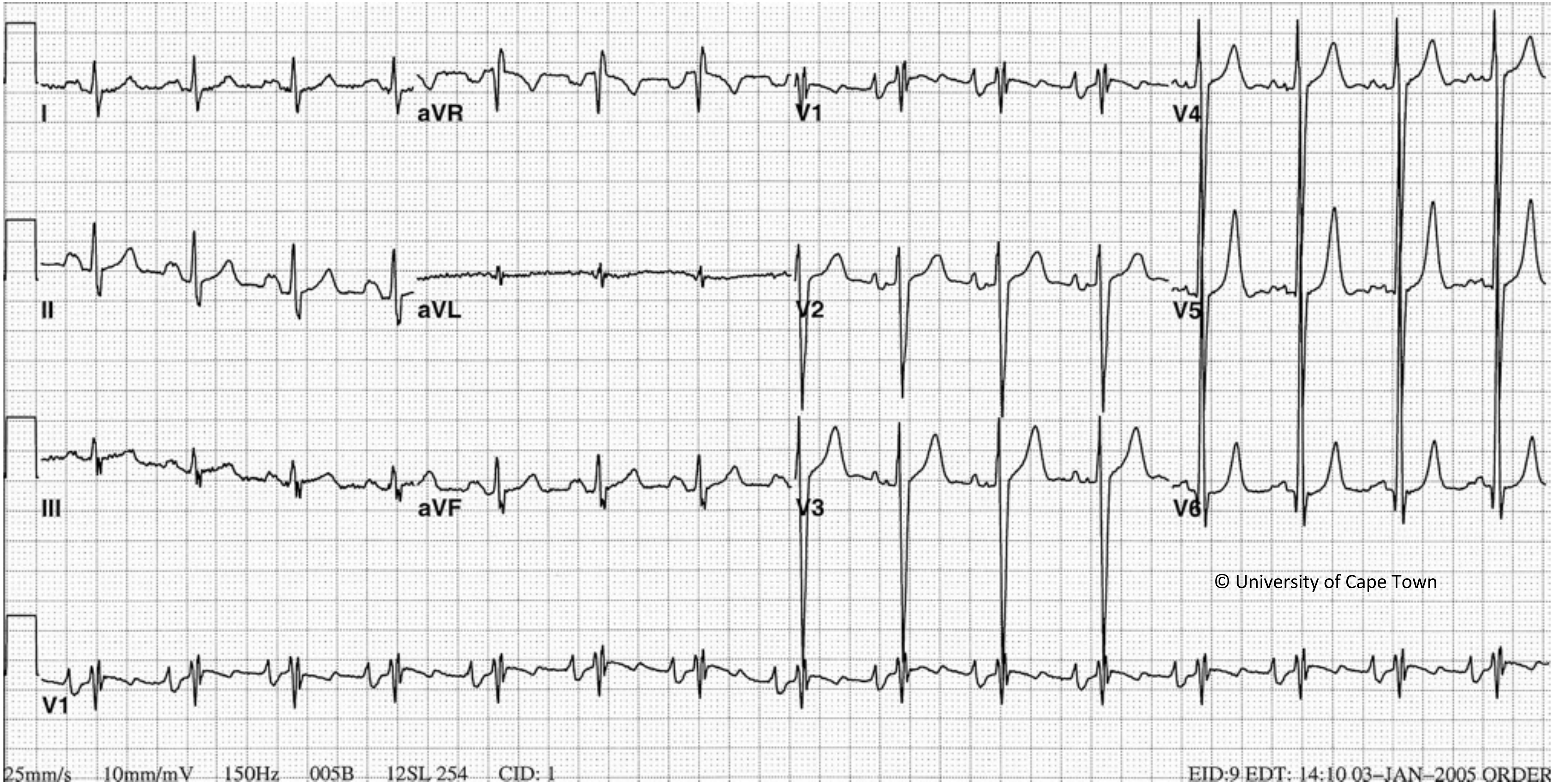

17. **An ECG is done during routine follow up at clinic of a 28 year old female patient with rheumatic aortic and mitral valve disease**.<br>What is the morphological abnormality on this ECG?	
	a. This is a normal ECG
x	b. Left atrial enlargement
	c. Right atrial enlargement
	d. Right ventricular hypertrophy (RVH)
	e. I do not know the answer

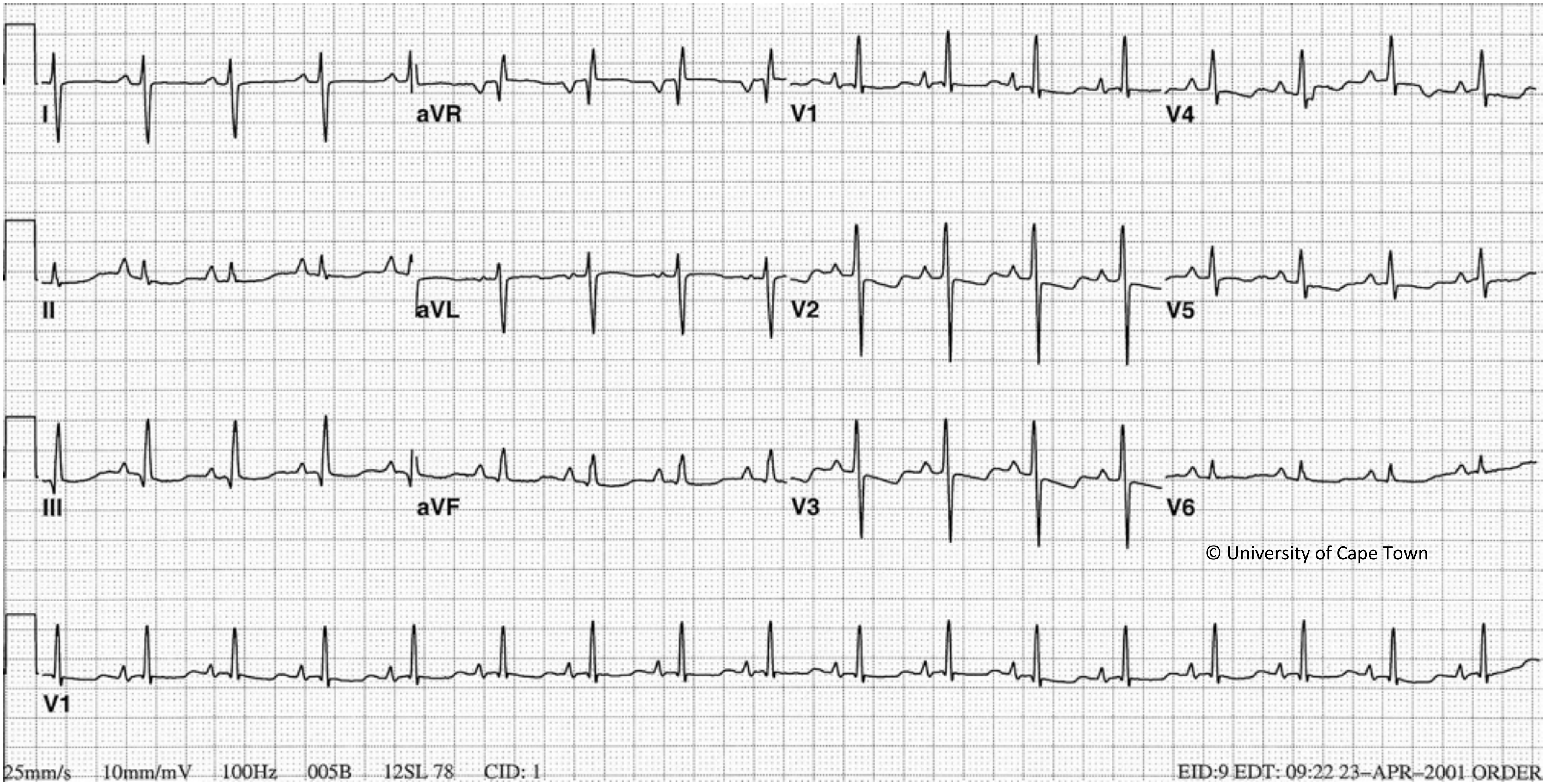

© University of Cape Town

18. **An ECG is done on a patient who has post TB bronchiectasis**.<br>Which of the following choices would describe an abnormality on this ECG?	
	a. This is a normal ECG
	b. Left atrial enlargement
x	c. Right atrial enlargement
	d. Left ventricular hypertrophy (LVH)
	e. I do not know the answer

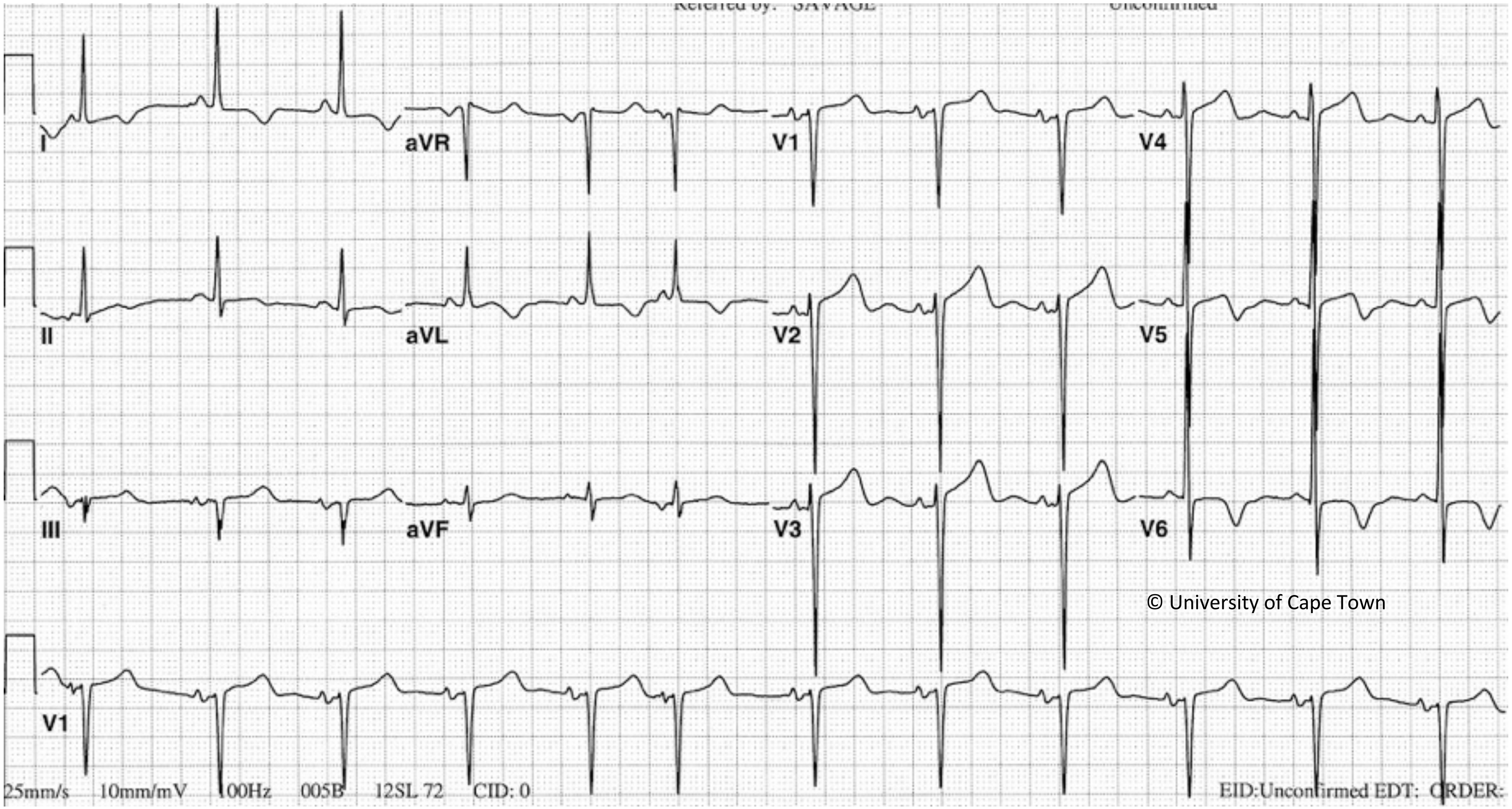

19. **An ECG is done on a 61 year old male patient with hypertension. The patient is asymptomatic.** What diagnosis can you make from this ECG?	
	a. Pericarditis
	b. ST-segment elevation myocardial infarction (STEMI)
x	c. Left ventricular hypertrophy (LVH) with repolarisation changes
	d. Right ventricular hypertrophy (RVH) with repolarisation changes
	e. I do not know the answer

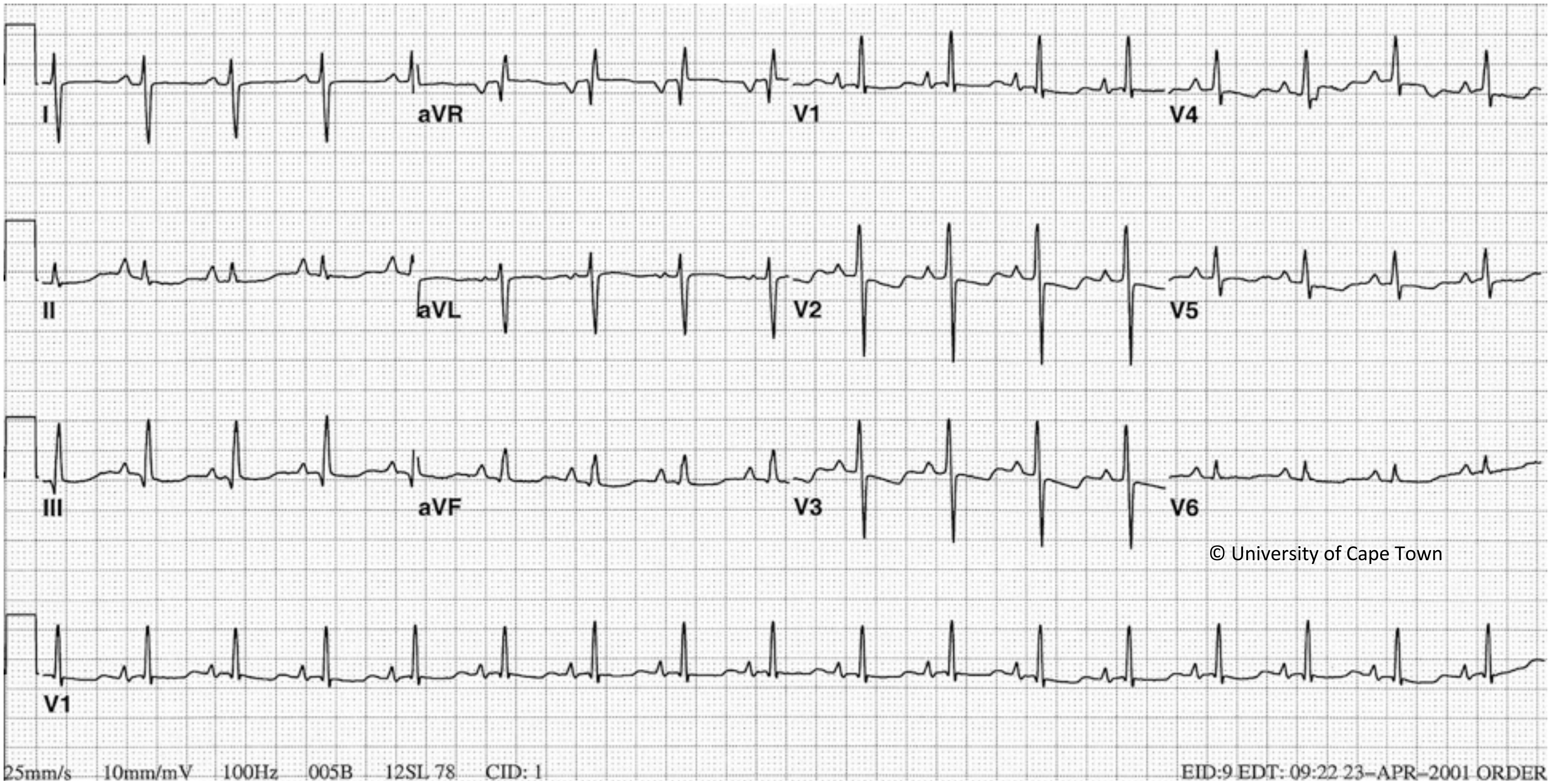

20. **An ECG is done on a 53 year man with COPD and cor pulmonale.**<br>What diagnosis can you make from this ECG?	
	a. Pericarditis
	b. ST-segment elevation myocardial infarction (STEMI)
	c. Left ventricular hypertrophy (LVH) with repolarisation changes
x	d. Right ventricular hypertrophy (RVH) with repolarisation changes
	e. I do not know the answer

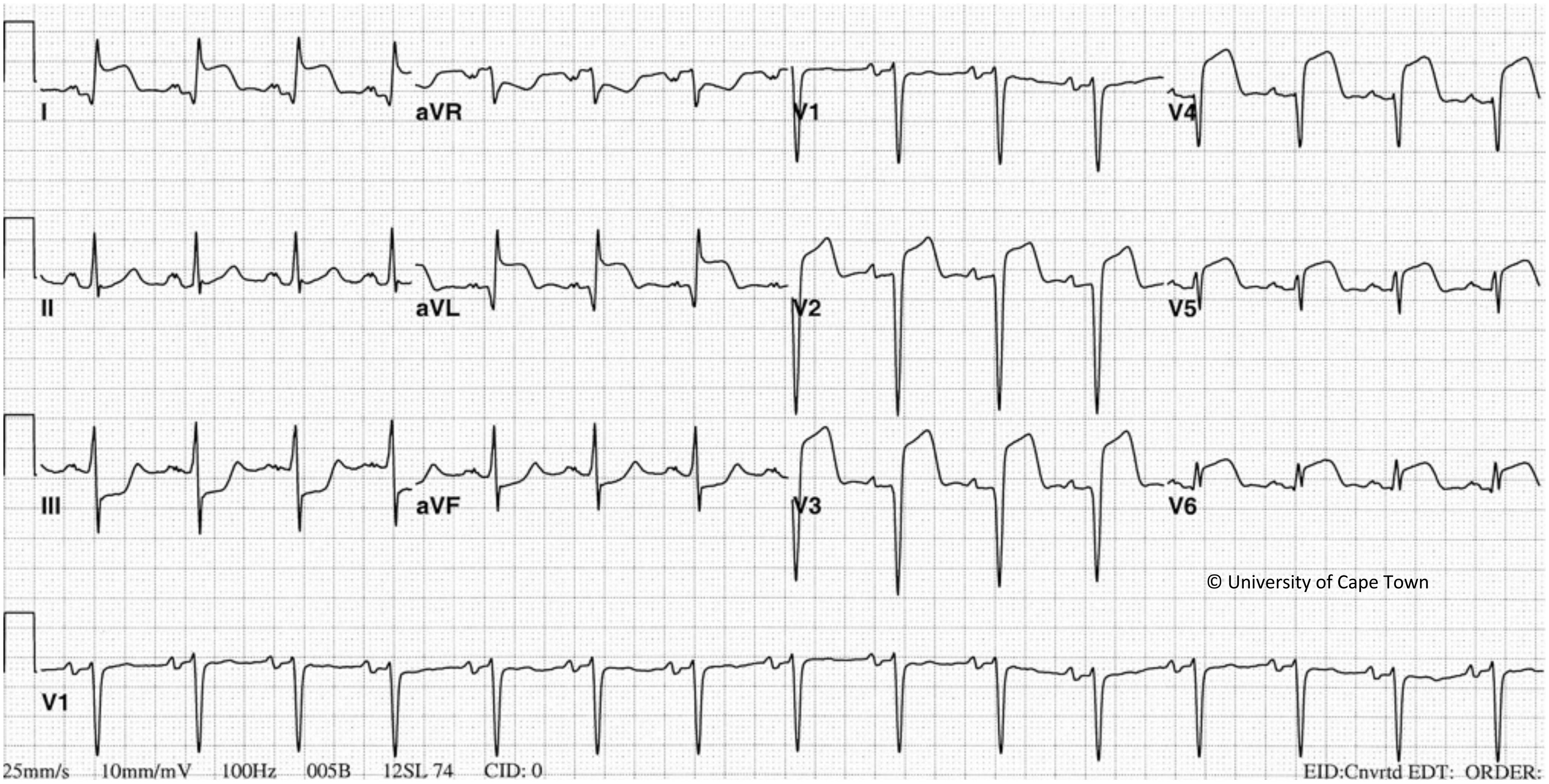

21. **A 55 year old man with hypertension, dyslipidaemia and 25 pack year smoking history presents with crushing retrosternal pain**.<br>What is your diagnosis?	
x	a. Antero-lateral ST-segment elevation myocardial infarction (STEMI)
	b. Inferior ST-segment elevation myocardial infarction (STEMI)
	c. Posterior ST-segment elevation myocardial infarction (STEMI)
	d. Pericarditis
	e. I do not know the answer

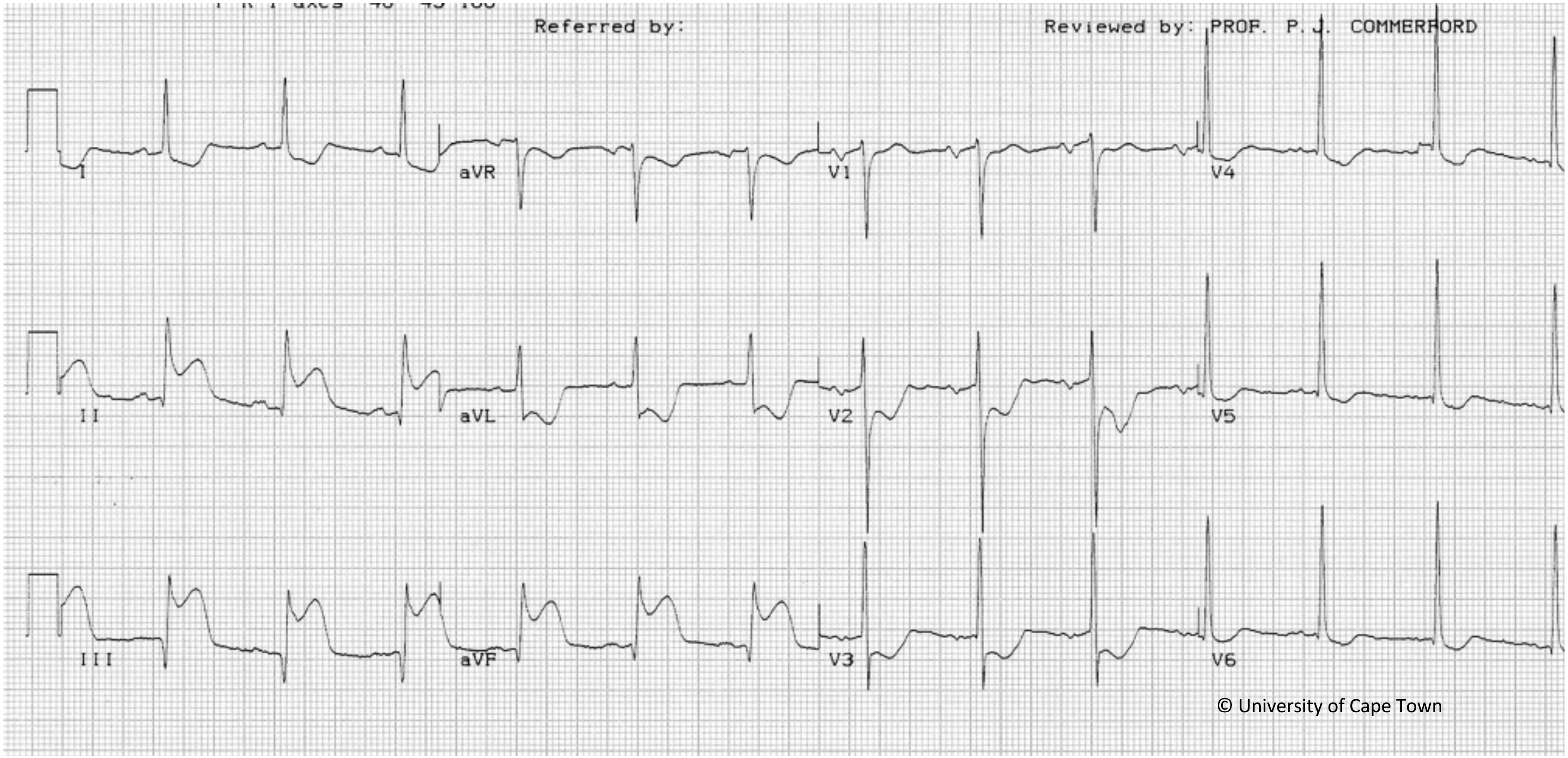

	22. **A 67 year old man with hypertension and diabetes presents with dull central chest pain, radiating down his left arm**.<br>What is your diagnosis?
	a. Anterior ST-segment elevation myocardial infarction (STEMI)
	b. Lateral ST-segment elevation myocardial infarction (STEMI)
x	c. Inferior ST-segment elevation myocardial infarction (STEMI) with posterior extension
	d. Posterior ST-segment elevation myocardial infarction (STEMI)
	e. I do not know the answer

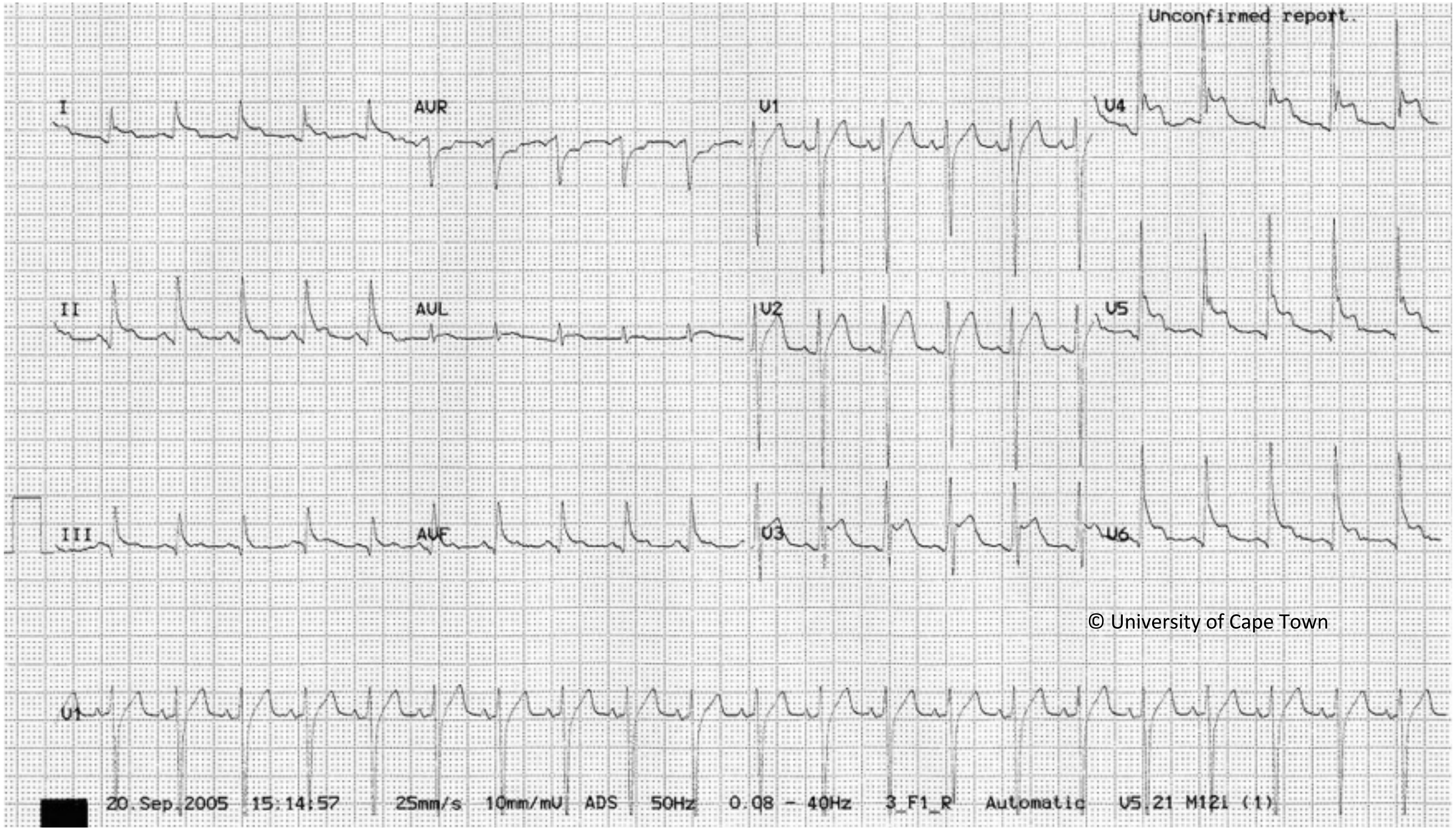

© University of Cape Town

23. **A 24 year old man with HIV, presents with stabbing chest pains, worsened by deep breaths and lying down**.<br>What is your diagnosis?	
	a. Anterolateral ST-segment elevation myocardial infarction (STEMI)
	b. Inferolateral ST-segment elevation myocardial infarction (STEMI)
	c. Non-ST-segment elevation myocardial infarction (NSTEMI)
x	d. Pericarditis
	e. I do not know the answer

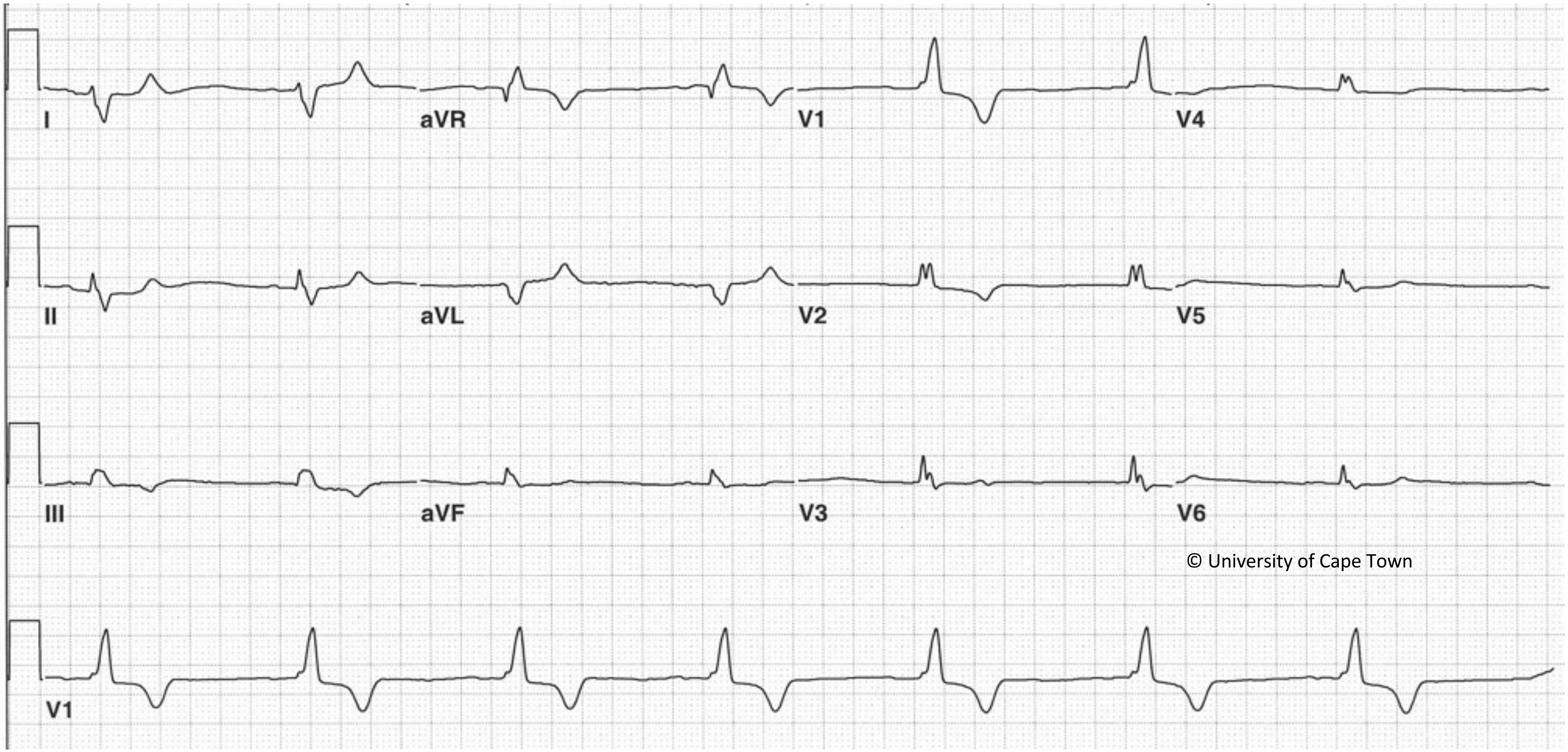

24. **A 58 year old male patient in the chronic dialysis unit is feeling lightheaded, weak and short of breath**.<br>What is your diagnosis?	
	a. Third degree AV block
	b. Sinus bradycardia
	c. Hypothermia
x	d. Hyperkalaemia
	e. I do not know the answer

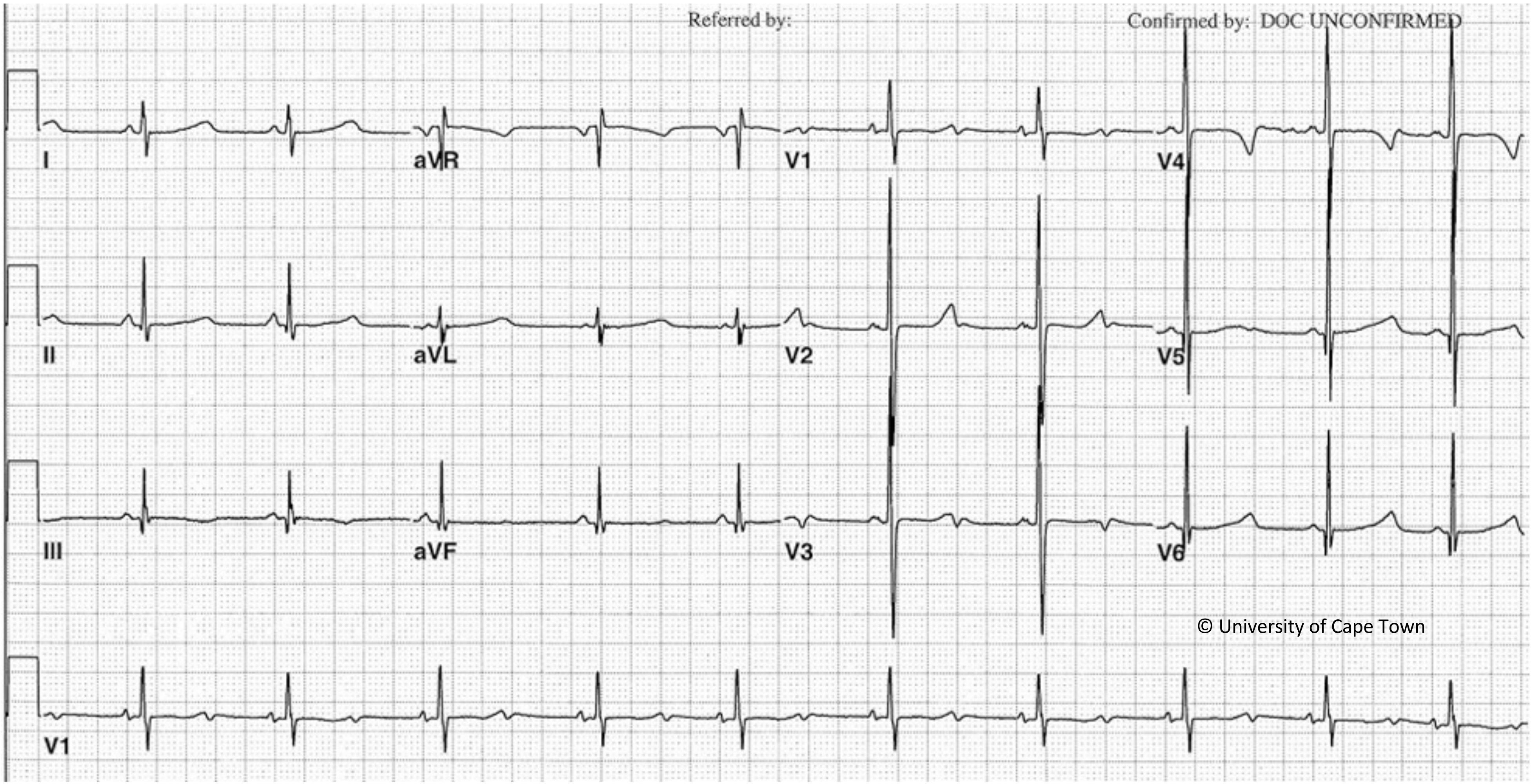

© University of Cape Town

25. **An ECG is done on a 72 year old female patient with previous thyroidectomy. She has cramps and is found to have hypocalcaemia.** What is the abnormality on this ECG?	
	a. This is a normal ECG
	b. 2:1 second degree AV block
x	c. Prolonged QT interval
	d. U waves
	e. I do not know the answer
